# Supplementary material for: On the role of asymmetric molecular geometry in high-performance organic solar cells
Source: Nat Commun. 2024 Apr 16;15:3287. doi: 10.1038/s41467-024-47707-5 (PMC11021434; doi:10.1038/s41467-024-47707-5)
Supplement: Supplementary file 1 — Supplementary Information [file 41467_2024_47707_MOESM1_ESM.pdf]

Supplementary Information

On the role of asymmetric molecular geometry in high-performance organic solar cells

Jinfeng Huang[1,2§], Tianyi Chen[1§], Le Mei[3,8§], Mengting Wang[1§], Yuxuan Zhu[4], Jiting Cui[5], Yanni Ouyang[6], Youwen Pan[1], Zhaozhao Bi[7], Wei Ma[7], Zaifei Ma[4], Haiming Zhu[5], Chunfeng Zhang[6], Xian-Kai Chen[8,9*], Hongzheng Chen[1*], Lijian Zuo[1,2*]

[1]State Key Laboratory of Silicon and Advanced Semiconductor Materials, Department of Polymer Science and Engineering, Zhejiang University, Hangzhou 310027, P. R. China.

[2]Zhejiang University-Hangzhou Global Scientific and Technological Innovation Center, Hangzhou 310014, P. R. China.

[3]Department of Chemistry, City University of Hong Kong, Kowloon 999077, Hong Kong.

[4]State Key Laboratory for Modification of Chemical Fibers and Polymer Materials, Center for Advanced Low-dimension Materials, College of Materials Science and Engineering, Donghua University, Shanghai 201620, China.

[5]State Key Laboratory of Modern Optical Instrumentation, Key Laboratory of Excited-State Materials of Zhejiang Province, Department of Chemistry, Zhejiang University, Hangzhou, Zhejiang 310027, China.

[6]National Laboratory of Solid State Microstructures, School of Physics, and Collaborative Innovation Center for Advanced Microstructures, Nanjing University, Nanjing 210093, China.

[7]State Key Laboratory for Mechanical Behavior of Materials, Xi'an Jiaotong University, Xi'an Jiaotong University, Xi'an 710049, P. R. China.

[8]Institute of Functional Nano & Soft Materials (FUNSOM), Soochow University, Suzhou 215123, Jiangsu, P. R. China.

[9]Jiangsu Key Laboratory of Advanced Negative Carbon Technologies, Soochow University, Suzhou 215123, Jiangsu, P. R. China.
[§]These authors contributed equally: Jinfeng Huang, Tianyi Chen, Le Mei, Mengting Wang.
[*]E-mail: zjuzlj@zju.edu.cn, hzchen@zju.edu.cn, xkchen@suda.edu.cn

Supplementary Table of Contents

1. Supplementary DFT Calculation

Supplementary Fig. 1 DFT calculation results of molecular orbitals of the Y-shape acceptors with chlorination of end groups on different positions at the B3LYP/6-31G(d) level.

Supplementary Fig. 2 The HOMO and LUMO energy level of the Y-shape acceptors with simplified side chains as methyl groups based on the DFT calculation at the long-range corrected ωB97XD/6-31G (d, p) level (e. g. SCl representing C11-BO-SCl, 2BO-SCl or BO-EH-SCl).

2. Supplementary Synthetic Routes and Characterization

Supplementary Fig. 3 Synthetic routes for the acceptors.

Supplementary Fig. 4 ^{1}H NMR spectrum of C11-BO-SF in $CDCl_3$.

Supplementary Fig. 5 MALDI-TOF mass spectra of C11-BO-SF.

Supplementary Fig. 6 ^{1}H NMR spectrum of C11-BO-SCl in $CDCl_3$.

Supplementary Fig. 7 MALDI-TOF mass spectra of C11-BO-SCl.

Supplementary Fig. 8 ^{1}H NMR spectrum of C11-BO-SBr in $CDCl_3$.

Supplementary Fig. 9 MALDI-TOF mass spectra of C11-BO-SBr.

Supplementary Fig. 10 ^{1}H NMR spectrum of 2BO-SCl in $CDCl_3$.

Supplementary Fig. 11 MALDI-TOF mass spectra of 2BO-SCl.

Supplementary Fig. 12 ^{1}H NMR spectrum of BO-EH-SCl in $CDCl_3$.

Supplementary Fig. 13 MALDI-TOF mass spectra of BO-EH-SCl.

Supplementary Fig. 14 ^{1}H NMR spectrum of BO-EH-SBr in $CDCl_3$.

Supplementary Fig. 15 MALDI-TOF mass spectra of BO-EH-SBr.

Supplementary Fig. 16 ^{1}H NMR spectrum of C11-BO-AF in $CDCl_3$.

Supplementary Fig. 17 MALDI-TOF mass spectra of C11-BO-AF.

Supplementary Fig. 18 ^{1}H NMR spectrum of C11-BO-ACl in $CDCl_3$.

Supplementary Fig. 19 MALDI-TOF mass spectra of C11-BO-ACl.

Supplementary Fig. 20 ^{1}H NMR spectrum of C11-BO-ABr in $CDCl_3$.

Supplementary Fig. 21 MALDI-TOF mass spectra of C11-BO-ABr.

Supplementary Fig. 22 ^{1}H NMR spectrum of 2BO-ACl in $CDCl_3$.

Supplementary Fig. 23 MALDI-TOF mass spectra of 2BO-ACl.

Supplementary Fig. 24 ^{1}H NMR spectrum of BO-EH-ACl in $CDCl_3$.

Supplementary Fig. 25 MALDI-TOF mass spectra of BO-EH-ACl.

Supplementary Fig. 26 ^{1}H NMR spectrum of BO-EH-ABr in $CDCl_3$.

Supplementary Fig. 27 MALDI-TOF mass spectra of BO-EH-ABr.

3. Supplementary Absorption and Energy Level Measurements

Supplementary Fig. 28 UV-vis curves of a C11-BO-SF and C11-BO-AF films, b C11-BO-SCl and C11-BO-ACl films, c C11-BO-SBr and C11-BO-ABr films, d 2BO-SCl and 2BO-ACl films, e BO-EH-SCl and BO-EH-ACl films and f BO-EH-SBr and BO-EH-ABr films.

Supplementary Fig. 29 CV curves of a C11-BO-SF and C11-BO-AF films, b C11-BO-SCl and C11-BO-ACl films, c C11-BO-SBr and C11-BO-ABr films, d BO-EH-SCl and BO-EH-ACl films, e 2BO-SCl and 2BO-ACl films and f BO-EH-SBr and BO-EH-ABr films.

4. Supplementary Morphology Characterization

Supplementary Fig. 30 AFM height and phase images of blend films with different acceptors.

Supplementary Fig. 31 2D GIWAXS images of a pristine acceptor films (including BO-EH-SCl and BO-EH-ACl) and b blend films (including PM6:BO-EH-SCl and PM6:BO-EH-ACl). c GIWAXS intensity profiles of the corresponding films along the in-plane and out-of-plane directions.

Supplementary Fig. 32 Contact angle images of PM6 and acceptors in thin films with water and diiodomethane droplet on top.

5. Supplementary Energy Loss Calculation

Supplementary Fig. 33 EQE curves of a C11-BO-SF and C11-BO-AF based films, b C11-BO-SCl and C11-BO-ACl based films, c C11-BO-SBr and C11-BO-ABr based films, d 2BO-SCl and 2BO-ACl based films, e BO-EH-SCl and BO-EH-ACl based films and f BO-EH-SBr and BO-EH-ABr based films.

Supplementary Fig. 34 Urbach Energy (E_U) calculation based on sEQE curves of a

BO-EH-SCl and b BO-EH-ACl based films.

Supplementary Fig. 35 E_g determination method based on absorption curves and PL curves of a PM6:BO-EH-SCl and b PM6:BO-EH-ACl based blend films.

6. Supplementary Charge Transfer, Transport and Recombination

Supplementary Fig. 36 PL spectra of a C11-BO-SF and C11-BO-AF based pure and blend films, b C11-BO-SCl and C11-BO-ACl based pure and blend films, c C11-BO-SBr and C11-BO-ABr based pure and blend films, d 2BO-SCl and 2BO-ACl based pure and blend films, e BO-EH-SCl and BO-EH-ACl based pure and blend films and f BO-EH-SBr and BO-EH-ABr based pure and blend films.

Supplementary Fig. 37 a–d 2D TAS spectra. e–h Color plots of the TAS spectra. i–l TA traces of the pure film for BO-EH-SCl and BO-EH-ACl.

Supplementary Fig. 38 a Electron and b hole mobility of the optimal devices.

7. Supplementary MD Simulation

Supplementary Fig. 39 a Interaction energy, and b contact probability after adjusting the two fragments based on the same atom numbers.

Supplementary Fig. 40 HOMO and LUMO of three typical complexes (D_0-A_1 or A_2 for PM6:BO-EH-SBr, A_0-A_1 and A_0-A_2 for PM6:BO-EH-ABr).

8. Supplementary Device Fabrication and Characterization

(1) Space Charge Limited Current (SCLC) Measurement

(2) Photoluminescence (PL) Measurement

(3) Electroluminescence External Quantum Efficiency (EQE_{EL})

(4) Electroluminescence (EL) Measurement

(5) Atomic Force Microscope (AFM) Measurement

(6) Transient Absorption Spectroscopy (TAS) Measurement

(7) Grazing Incidence Wide-Angle X-ray Scattering (GIWAXS) Characterization

9. Supplementary Molecular Dynamics Simulations Method

10. Supplementary Table

Supplementary Table 1 Comparison of efficiency and V_{oc} for binary OPVs without special treatment between this work and references.

Supplementary Table 2 DFT, UV-vis and CV results of pure acceptors.

Supplementary Table 3 Detailed data of morphology characterizations.
Supplementary Table 4 Characterizations of charge transfer, transport and recombination.
Supplementary Table 5 Average ESP of each atom of five fragments calculated on B3LYP/6-31G (d, p) level.
11. Supplementary References

1. Supplementary DFT Calculation

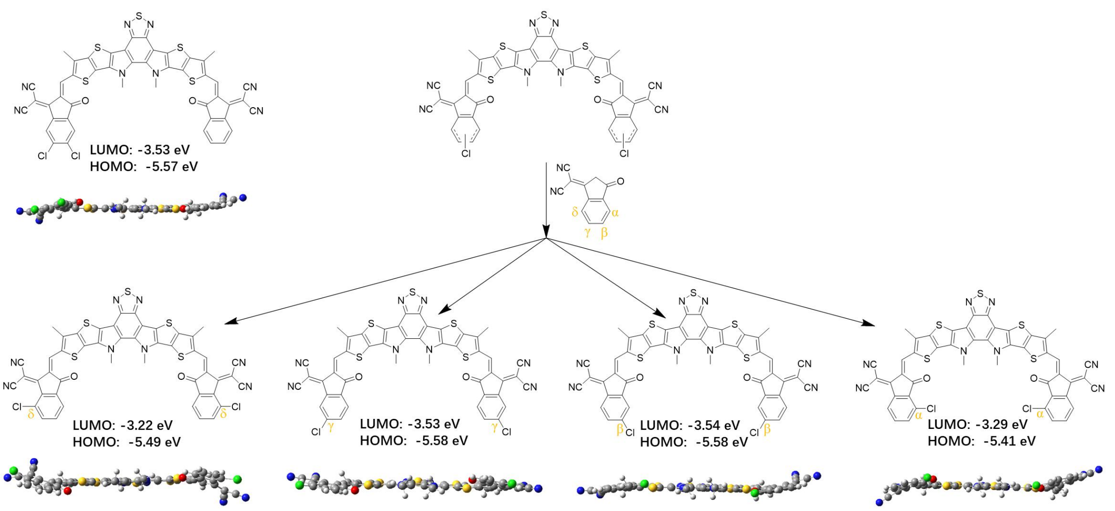

Supplementary Fig. 1 DFT calculation results of molecular orbitals of the Y-shape acceptors with chlorination of end groups on different positions at the B3LYP/6-31G(d) level.

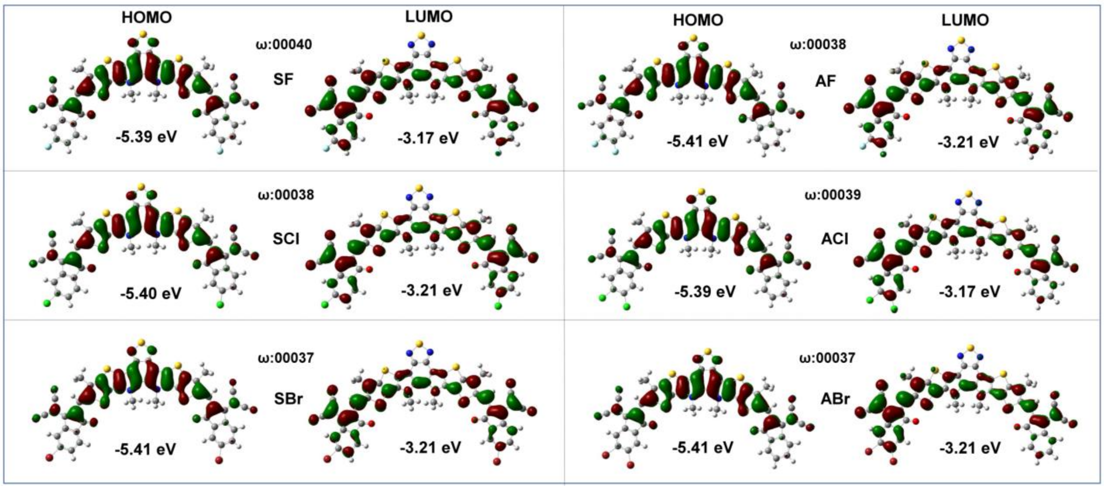

Supplementary Fig. 2 The HOMO and LUMO energy level of the Y-shape acceptors with simplified side chains as methyl groups based on the DFT calculation at the long-range corrected ωB97XD/6-31G (d, p) level (e. g. SCl representing C11-BO-SCl, 2BO-SCl or BO-EH-SCl).

2. Supplementary Synthetic Routes and Characterization

All reagents and solvents, unless otherwise specified, were purchased from commercial sources and were used without further purification.

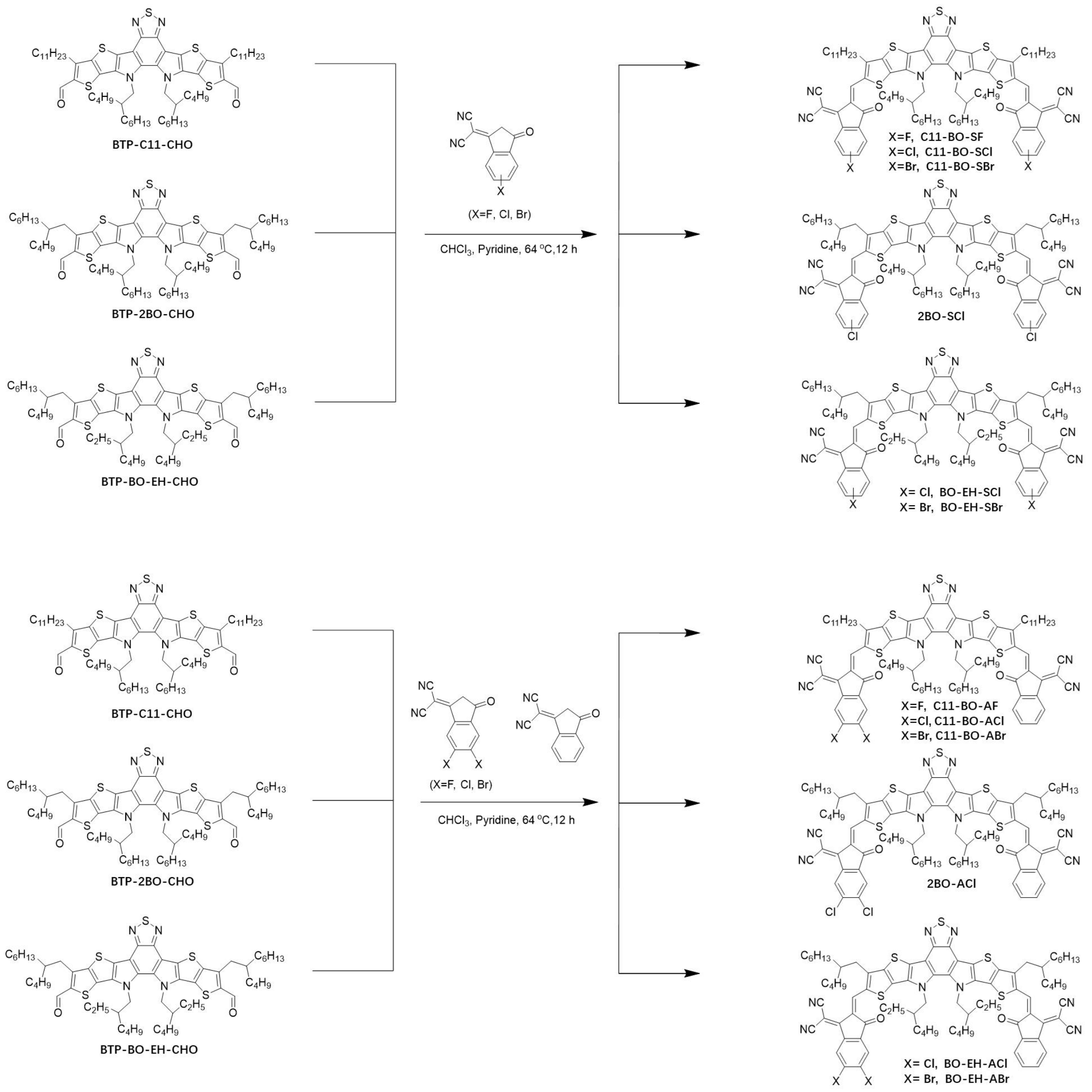

Supplementary Fig. 3 Synthetic routes for the acceptors.

(1) C11-BO-SF

BTP-C11-CHO(100 mg, 0.09 mmol), 2-(5 or

6-fluoro-3-oxo-2,3-dihydro-1H-inden-1-ylidene)malononitrile (57 mg, 0.27 mmol) and $CHCl_3$ (20mL) were added to a Schlenk tube under the protection of Argon. The mixture was stirred at 64 °C for 30 mins, and then 0.2 mL pyridine was added. After the mixture was refluxed for 12 h, the solvent was removed and silica gel column chromatography was used to purify the product with the mixture of chloroform and petroleum ether (1:1~1:1.2, v/v) as the eluent, yielding a black solid (91 mg, 68%). ^1H NMR (500 MHz, $CDCl_3$, δ): 9.17 (d, J = 7.0 Hz, 2H), 8.73-8.71 (m, 0.7 H), 8.40-8.37 (m, 1.3 H), 7.96-7.93 (m, 1.3 H), 7.59-7.57 (m, 0.7 H), 7.45-7.41 (m, 2H), 4.78 (d, J = 8.2 Hz, 4H), 3.22 (d, J = 12.1 Hz, 4H), 2.15 (s, 2H), 1.91-1.86 (m, 4H), 1.56-1.49 (m, 6H), 1.38 (t, J = 7.2 Hz, 4H), 1.30-0.85 (m, 60H), 0.72-0.63 (m, 12H). MS (MALDI-TOF): Calcd for $C_{90}H_{104}F_2N_8O_2S_5$ (M^+): 1526.69, Found: 1527.56.

(2) C11-BO-SCl

BTP-C11-CHO (100 mg, 0.09 mmol), 2-(5 or 6-chloro-3-oxo-2,3-dihydro-1H-inden-1-ylidene)malononitrile (61 mg, 0.27 mmol) and $CHCl_3$ (20mL) were added to a Schlenk tube under the protection of Argon. The mixture was stirred at 64 °C for 30 mins, and then 0.2 mL pyridine was added. After the mixture was refluxed for 12 h, the solvent was removed and silica gel column chromatography was used to purify the product with the mixture of chloroform and petroleum ether (1:1~1:1.2, v/v) as the eluent, yielding a black solid (114 mg, 82%). ^1H NMR (500 MHz, $CDCl_3$, δ): 9.18 (s, 2H), 8.68-8.63 (m, 2H), 7.87 (t, J = 3.8 Hz, 2H), 7.72-7.69 (m, 2H), 4.77 (s, 4H), 3.22 (s, 4H), 2.14 (d, 2H), 1.90 (m, 4H), 1.54-1.48 (m, 6H), 1.39-1.34 (m, 4H), 1.31-0.85 (m, 60H), 0.71-0.63 (m, 12H). MS (MALDI-TOF): Calcd for $C_{90}H_{104}Cl_2N_8O_2S_5$ (M^+): 1558.63, Found: 1560.58.

(3) C11-BO-SBr

BTP-C11-CHO (100 mg, 0.09 mmol), 2-(5 or 6-bromo-3-oxo-2,3-dihydro-1H-inden-1-ylidene)malononitrile (71 mg, 0.27 mmol) and $CHCl_3$ (20mL) were added to a Schlenk tube under the protection of Argon. The mixture was stirred at 64 °C for 30 mins, and then 0.2 mL pyridine was added. After

the mixture was refluxed for 12 h, the solvent was removed and silica gel column chromatography was used to purify the product with the mixture of chloroform and petroleum ether (1:1~1:1.2, v/v) as the eluent, yielding a black solid (112 mg, 78%). ^{1}H NMR (500 MHz, $CDCl_3$, δ): 9.16 (t, J = 4.7 Hz, 2H), 8.82 (t, J = 3.2 Hz, 2H), 8.55-8.52 (m, 2H), 8.03 (d, J = 1.9 Hz, 4H), 7.89-7.87 (m, 2H), 7.86-7.83 (m, 2 H), 7.80 (d, J = 7.4 Hz, 4H), 4.79 (t, J = 5.1 Hz, 4H), 3.23 (s, 4H), 2.16 (d, J = 7.5 Hz, 2H), 1.89 (t, J = 7.7 Hz, 4H), 1.55-1.47 (m, 6H), 1.37 (s, 4H), 1.31-0.85 (m, 60H), 0.71-0.65 (m, 12H). MS (MALDI-TOF): Calcd for $C_{90}H_{104}Br_2N_8O_2S_5$ (M^+): 1646.53, Found: 1648.53.

(4) 2BO-SCl

BTP-2BO-CHO (100 mg, 0.09 mmol), 2-(5 or 6-chloro-3-oxo-2,3-dihydro-1H-inden-1-ylidene)malononitrile (61 mg, 0.27 mmol) and CHCl3 (20mL) were added to a Schlenk tube under the protection of Argon. The mixture was stirred at 64 °C for 30 mins, and then 0.2 mL pyridine was added. After the mixture was refluxed for 12 h, the solvent was removed and silica gel column chromatography was used to purify the product with the mixture of chloroform and petroleum ether (1:1~1:1.2, v/v) as the eluent, yielding a black solid (117 mg, 87%). ^{1}H NMR (500 MHz, $CDCl_3$, δ): 9.17 (s, 2H), 8.69 (d, J = 1.6 Hz, 1.2H), 8.65 (d, J = 8.4 Hz, 0.8H), 7.87-7.85 (m, 2H), 7.72-7.69 (m, 2 H), 4.78 (d, J = 6.2 Hz, 4H), 3.19 (d, J = 7.6 Hz, 4H), 2.14-2.08 (m, 4H), 1.57 (s, 4H), 1.47-0.83 (m, 72H), 0.70-0.63 (m, 12H). MS (MALDI-TOF): Calcd for $C_{92}H_{108}Cl_2N_8O_2S_5$ (M^+): 1586.66, Found: 1582.79.

(5) BO-EH-SCl

BTP-BO-EH-CHO (100 mg, 0.09 mmol), 2-(5 or 6-chloro-3-oxo-2,3-dihydro-1H-inden-1-ylidene)malononitrile (61 mg, 0.27 mmol) and $CHCl_3$ (20mL) were added to a Schlenk tube under the protection of Argon. The mixture was stirred at 64 °C for 30 mins, and then 0.2 mL pyridine was added. After the mixture was refluxed for 12 h, the solvent was removed and silica gel column

chromatography was used to purify the product with the mixture of chloroform and petroleum ether (1:1~1:1.2, v/v) as the eluent, yielding a black solid (115 mg, 83%). ^{1}H NMR (500 MHz, $CDCl_3$, δ): 9.16 (s, 2H), 8.68 (d, *J* = 1.6 Hz, 1.2H), 8.65 (d, *J* = 8.4 Hz, 0.8H), 7.88-7.86 (m, 2H), 7.72-7.69 (m, 2 H), 4.78 (s, 4H), 3.18 (d, *J* = 7.6 Hz, 4H), 2.12-2.08 (m, 4H), 1.57 (s, 4H), 1.47-1.01 (m, 44H), 0.87-0.82 (m, 12H), 0.78-0.74 (m, 6H), 0.68-0.64 (m, 6H). MS (MALDI-TOF): Calcd for $C_{84}H_{92}Cl_2N_8O_2S_5$ (M^+): 1474.53, Found: 1473.34.

(6) BO-EH-SBr

BTP-BO-EH-CHO (100 mg, 0.09 mmol), 2-(5 or 6-bromo-3-oxo-2,3-dihydro-1H-inden-1-ylidene)malononitrile (71 mg, 0.27 mmol) and $CHCl_3$ (20mL) were added to a Schlenk tube under the protection of Argon. The mixture was stirred at 64 °C for 30 mins, and then 0.2 mL pyridine was added. After the mixture was refluxed for 12 h, the solvent was removed and silica gel column chromatography was used to purify the product with the mixture of chloroform and petroleum ether (1:2~1:2.2, v/v) as the eluent, yielding a black solid (115 mg, 78%). ^{1}H NMR (500 MHz, $CDCl_3$, δ): 9.19 (s, 2H), 8.88 (d, *J* = 1.2 Hz, 1.3H), 8.60 (d, *J* = 6.9 Hz, 0.7H), 8.08 (t, *J* = 1.5 Hz, 4H), 7.91-7.88 (m, 2 H), 7.83-7.81 (m, 1.3 H), 4.81 (d, *J* = 6.2 Hz, 2H), 3.22 (d, *J* = 6.3 Hz, 4H), 2.12 (d, *J* = 5.2 Hz, 4H), 1.57 (s, 4H), 1.51-1.16 (m, 44H), 0.90-0.84 (m, 12H), 0.80-0.76 (m, 6H), 0.68-0.65 (m, 6H). MS (MALDI-TOF): Calcd for $C_{84}H_{92}Br_2N_8O_2S_5$ (M^+): 1562.43, Found: 1565.43.

(7) C11-BO-AF

BTP-C11-CHO (300 mg, 0.26 mmol), 2-(5,6-difluoro-3-oxo-2,3-dihydro-1H-inden-1-ylidene)malononitrile (179 mg, 0.78 mmol), 2-(3-oxo-2,3-dihydro-1H-inden-1-ylidene)malononitrile (151 mg, 0.78 mmol) and $CHCl_3$ (60mL) were added to a Schlenk tube under the protection of Argon. The mixture was stirred at 64 °C for 30 mins, and then 0.6 mL pyridine was added. After the mixture was refluxed for 12 h, the solvent was removed and silica gel column chromatography was used to purify the product with the mixture of chloroform and

petroleum ether (1:1~1:1.2, v/v) as the eluent, yielding a black solid (128 mg, 32%).^{1}H NMR (500 MHz, $CDCl_3$, δ): 9.18 (d, J = 11.4 Hz, 2H), 8.73-8.71 (m, 1H), 8.58-8.55 (m, 1H), 7.96-7.95 (m, 1H), 7.80-7.77 (m, 2 H), 7.76-7.68 (m, 1H), 4.77 (d, J = 7.3 Hz, 4H), 3.24 (d, J = 1.5 Hz, 4H), 2.14 (s, 2H), 1.90-1.87 (m, 4H), 1.55-1.50 (m, 6H), 1.39 (t, J = 7.0 Hz, 4H), 1.31-0.85 (m, 60H), 0.72-0.64 (m, 12H). MS (MALDI-TOF): Calcd for $C_{90}H_{104}F_2N_8O_2S_5$ (M^+): 1526.69, Found: 1527.61.

(8) C11-BO-ACl

BTP-C11-CHO (300 mg, 0.26 mmol), 2-(5,6-dichloro-3-oxo-2,3-dihydro-1H-inden-1-ylidene)malononitrile (205 mg, 0.78 mmol), 2-(3-oxo-2,3-dihydro-1H-inden-1-ylidene)malononitrile (151 mg, 0.78 mmol) and $CHCl_3$ (20mL) were added to a Schlenk tube under the protection of Argon. The mixture was stirred at 64 °C for 30 mins, and then 0.2 mL pyridine was added. After the mixture was refluxed for 12 h, the solvent was removed and silica gel column chromatography was used to purify the product with the mixture of chloroform and petroleum ether (1:1~1:1.2, v/v) as the eluent, yielding a black solid (124 mg, 31%). ^{1}H NMR (500 MHz, $CDCl_3$, δ): 9.17 (s, 2H), 8.78 (s, 1H), 8.73-8.71 (s, 1H), 7.96-7.95 (m, 2H), 7.78-7.76 (m, 2 H), 4.78 (d, J = 7.4 Hz, 4H), 3.24 (d, J = 8.0 Hz, 4H), 2.16 (t, J = 6.9 Hz, 2H), 1.89 (d, J = 5.7 Hz, 4H), 1.55-1.51 (m, 6H), 1.37 (s, 4H), 1.30-0.85 (m, 60H), 0.72-0.64 (m, 12H). MS (MALDI-TOF): Calcd for $C_{90}H_{104}Cl_2N_8O_2S_5$ (M^+): 1558.63, Found: 1560.62.

(9) C11-BO-ABr

BTP-C11-CHO (300 mg, 0.26 mmol), 2-(5,6-dibromo-3-oxo-2,3-dihydro-1H-inden-1-ylidene)malononitrile (273 mg, 0.78 mmol), 2-(3-oxo-2,3-dihydro-1H-inden-1-ylidene)malononitrile (151 mg, 0.78 mmol) and $CHCl_3$ (20mL) were added to a Schlenk tube under the protection of Argon. The mixture was stirred at 64 °C for 30 mins, and then 0.2 mL pyridine was added. After the mixture was refluxed for 12 h, the solvent was removed and silica gel column chromatography was used to purify the product with the mixture of chloroform and

petroleum ether (1:1~1:1.2, v/v) as the eluent, yielding a black solid (121 mg, 28%). ^{1}H NMR (500 MHz, $CDCl_3$, δ): 9.18 (d, *J* = 10.6 Hz, 2H), 8.92 (s, 1H), 8.72-8.70 (m, 1H), 8.11 (s, 1H), 7.96-7.95 (m, 2H), 7.78-7.76 (m, 2H), 4.78 (d, *J* = 7.6 Hz, 4H), 3.25-3.20 (m, 4H), 1.90-1.85 (m, 2H), 1.55-1.49 (m, 4H), 1.37-1.26 (m, 6H), 1.37 (d, *J* = 11.1 Hz, 4H), 1.30-0.85 (m, 60H), 0.72-0.63 (m, 12H). MS (MALDI-TOF): Calcd for $C_{90}H_{104}Br_2N_8O_2S_5$ (M^+): 1646.53, Found: 1648.59.

(10) 2BO-ACl

BTP-2BO-CHO (300 mg, 0.26 mmol), 2-(5,6-dichloro-3-oxo-2,3-dihydro-1H-inden-1-ylidene)malononitrile (205 mg, 0.78 mmol), 2-(3-oxo-2,3-dihydro-1H-inden-1-ylidene)malononitrile (151 mg, 0.78 mmol) and $CHCl_3$ (20mL) were added to a Schlenk tube under the protection of Argon. The mixture was stirred at 64 °C for 30 mins, and then 0.2 mL pyridine was added. After the mixture was refluxed for 12 h, the solvent was removed and silica gel column chromatography was used to purify the product with the mixture of chloroform and petroleum ether (1:1~1:1.2, v/v) as the eluent, yielding a black solid (101 mg, 25%). ^{1}H NMR (500 MHz, $CDCl_3$, δ): 9.17 (s, 2H), 8.80 (s, 1H), 8.74-8.72 (m, 1H), 7.96-7.94 (m, 2H), 7.79-7.76 (m, 2 H), 4.78 (d, *J* = 7.9 Hz, 4H), 3.20 (t, *J* = 7.8 Hz, 4H), 2.13 (t, *J* = 10.7 Hz, 4H), 1.55 (s, 4H), 1.47-0.83 (m, 72H), 0.70-0.62 (m, 12H). MS (MALDI-TOF): Calcd for $C_{92}H_{108}Cl_2N_8O_2S_5$ (M^+): 1586.66, Found: 1582.84.

(11) BO-EH-ACl

BTP-BO-EH-CHO (300 mg, 0.28 mmol), 2-(5,6-dichloro-3-oxo-2,3-dihydro-1H-inden-1-ylidene)malononitrile (220 mg, 0.84 mmol), 2-(3-oxo-2,3-dihydro-1H-inden-1-ylidene)malononitrile (162 mg, 0.84 mmol) and $CHCl_3$ (20mL) were added to a Schlenk tube under the protection of Argon. The mixture was stirred at 64 °C for 30 mins, and then 0.2 mL pyridine was added. After the mixture was refluxed for 12 h, the solvent was removed and silica gel column chromatography was used to purify the product with the mixture of chloroform and petroleum ether (1:1~1:1.2, v/v) as the eluent, yielding a black solid (104 mg, 25%).

^{1}H NMR (500 MHz, $CDCl_3$, δ): 9.16 (s, 2H), 8.78 (s, 1H), 8.73-8.71 (m, 1H), 7.96-7.95 (m, 2H), 7.78-7.74 (m, 2 H), 4.79 (d, *J* = 7.8 Hz, 4H), 3.16 (s, 4H), 2.10 (d, *J* = 7.8 Hz, 4H), 1.56 (s, 4H), 1.47-0.98 (m, 44H), 0.88-0.82 (m, 12H), 0.79-0.74 (m, 6H), 0.68-0.64 (m, 6H). MS (MALDI-TOF): Calcd for $C_{84}H_{92}Cl_2N_8O_2S_5$ (M^+): 1474.53, Found: 1472.34.

(12) BO-EH-ABr

BTP-BO-EH-CHO (300 mg, 0.28 mmol), 2-(5,6-dibromo-3-oxo-2,3-dihydro-1H-inden-1-ylidene)malononitrile (294 mg, 0.84 mmol), 2-(3-oxo-2,3-dihydro-1H-inden-1-ylidene)malononitrile (162 mg, 0.84 mmol) and $CHCl_3$ (20mL) were added to a Schlenk tube under the protection of Argon. The mixture was stirred at 64 °C for 30 mins, and then 0.2 mL pyridine was added. After the mixture was refluxed for 12 h, the solvent was removed and silica gel column chromatography was used to purify the product with the mixture of chloroform and petroleum ether (1:2~1:3, v/v) as the eluent, yielding a black solid (93 mg, 21%). ^{1}H NMR (500 MHz, $CDCl_3$, δ): 9.19 (d, *J* = 11.6 Hz, 1.2H), 8.95 (s, 1H), 8.75 (d, *J* = 6.1 Hz, 0.8H), 8.14 (s, 1H), 7.99 (t, *J* = 3.2 Hz, 4H), 7.80-7.78 (m, 2 H), 4.81 (d, *J* = 7.6 Hz, 4H), 3.22 (m, 4H), 2.14-2.10 (m, 4H), 1.55 (s, 4H), 1.51-0.97 (m, 44H), 0.90-0.84 (m, 12H), 0.82-0.76 (m, 6H), 0.70-0.65 (m, 6H). MS (MALDI-TOF): Calcd for $C_{84}H_{92}Br_2N_8O_2S_5$ (M^+): 1562.43, Found: 1565.43.

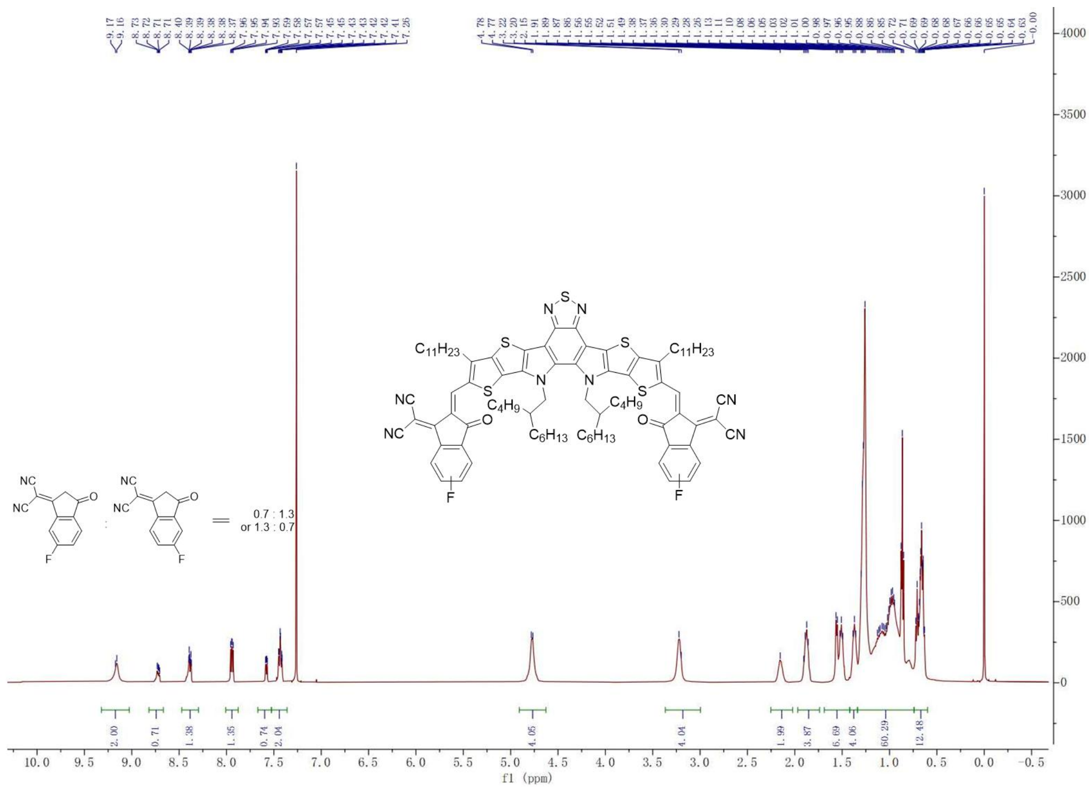

Supplementary Fig. 4 ^{1}H NMR spectrum of C11-BO-SF in $CDCl_3$.

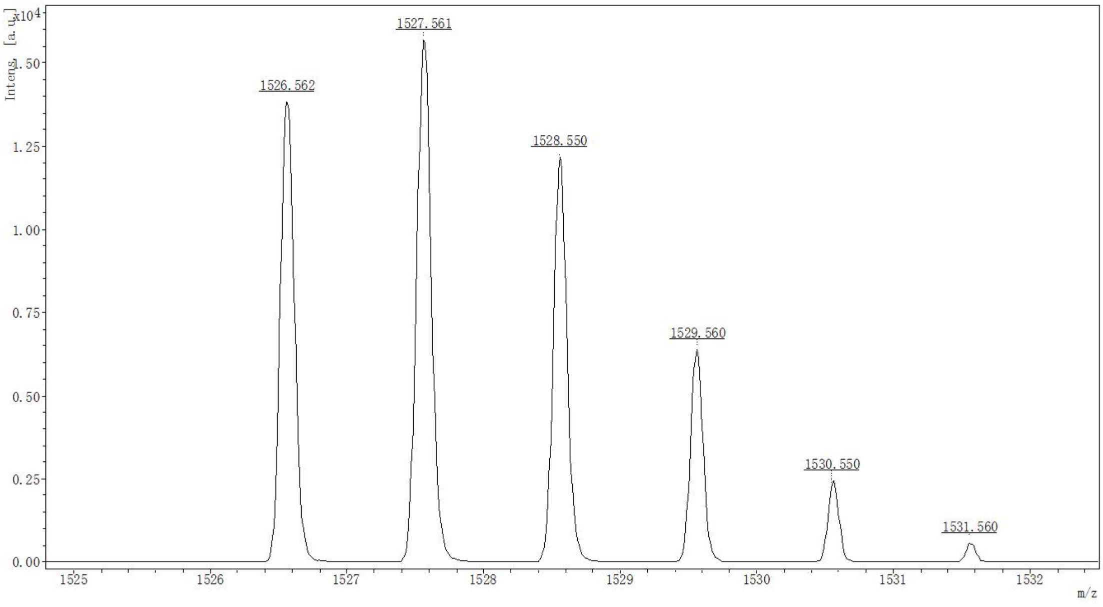

Supplementary Fig. 5 MALDI-TOF mass spectra of C11-BO-SF.

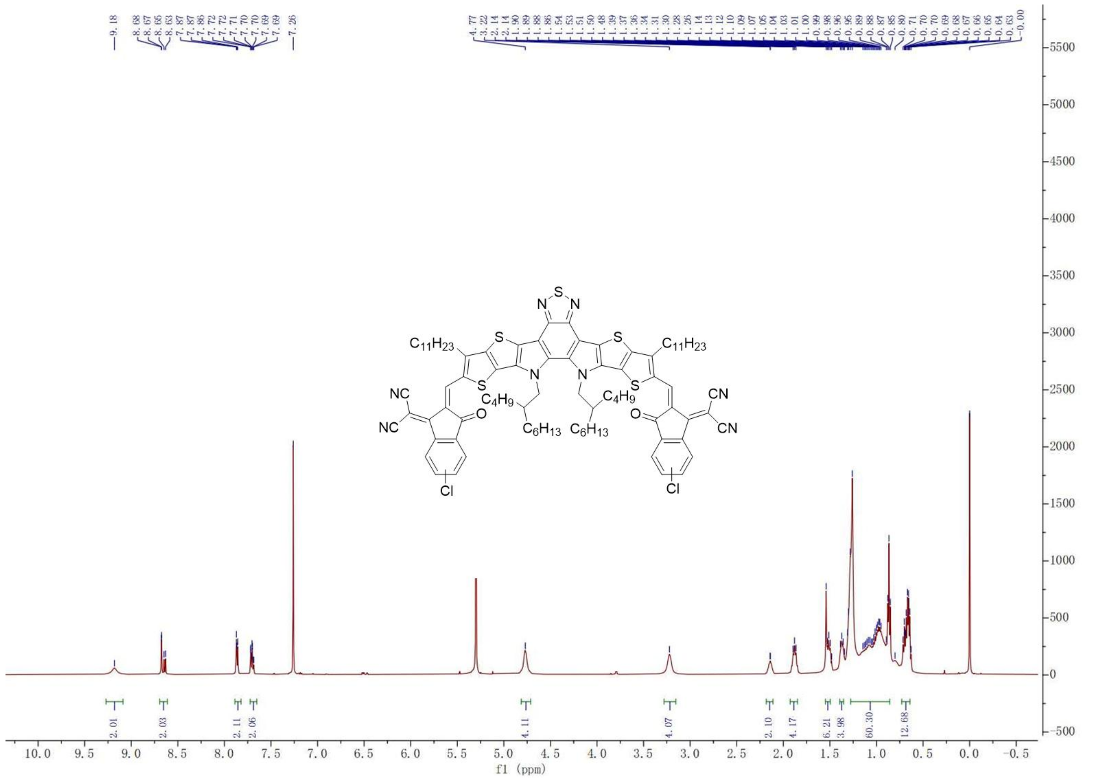

Supplementary Fig. 6 ^{1}H NMR spectrum of C11-BO-SCl in $CDCl_3$.

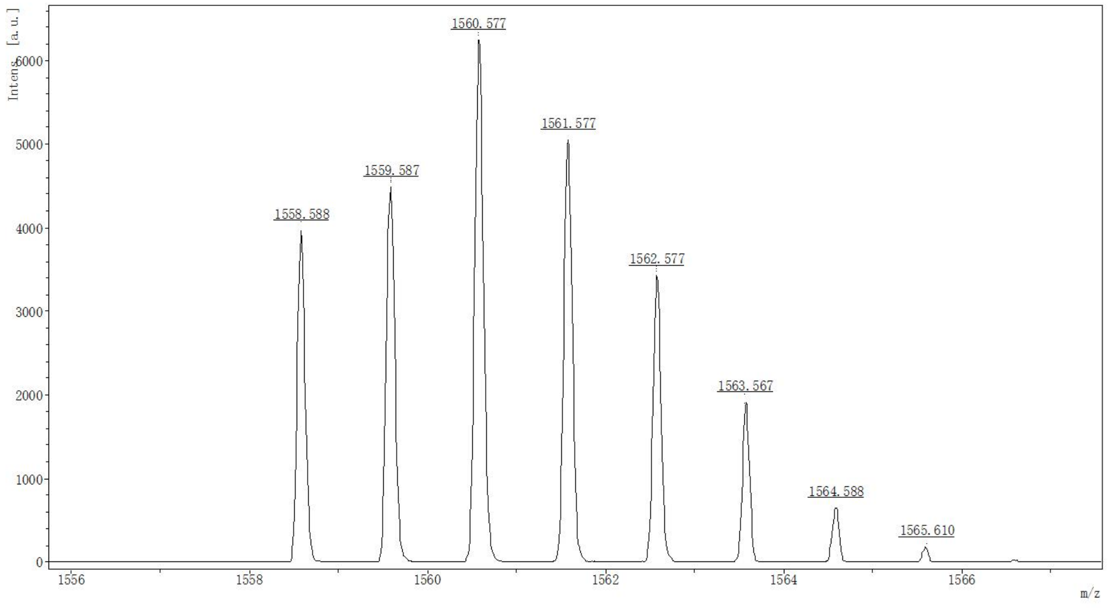

Supplementary Fig. 7 MALDI-TOF mass spectra of C11-BO-SCl.

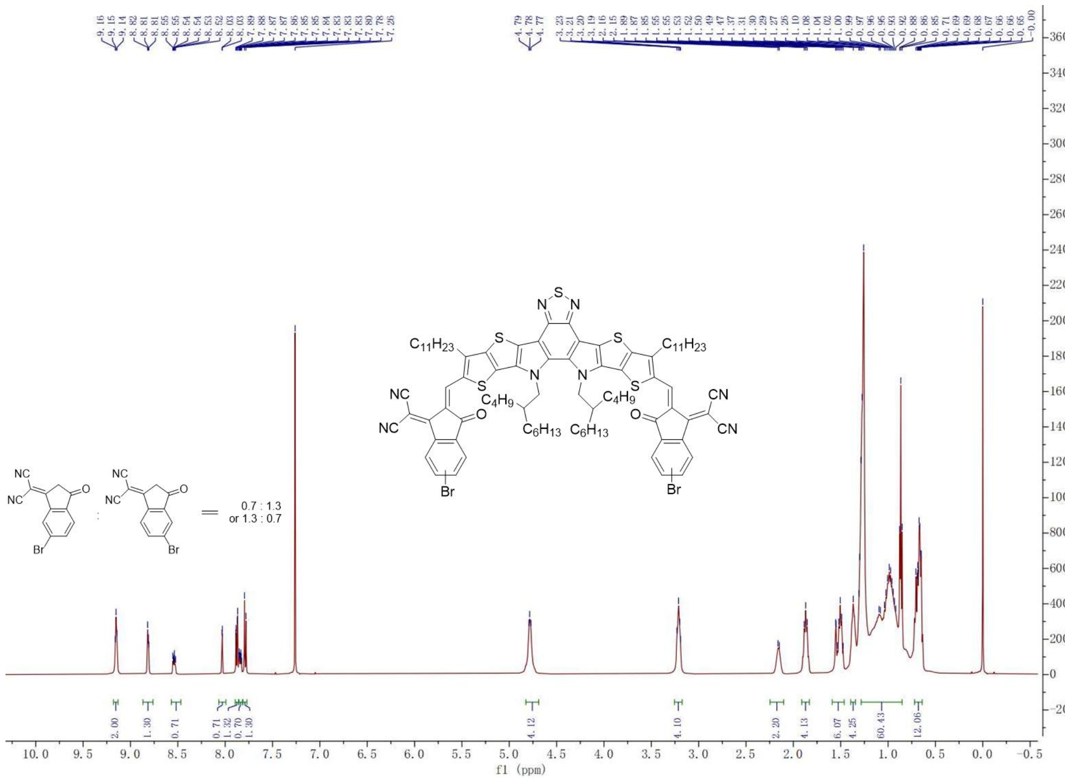

Supplementary Fig. 8 ^{1}H NMR spectrum of C11-BO-SBr in $CDCl_3$.

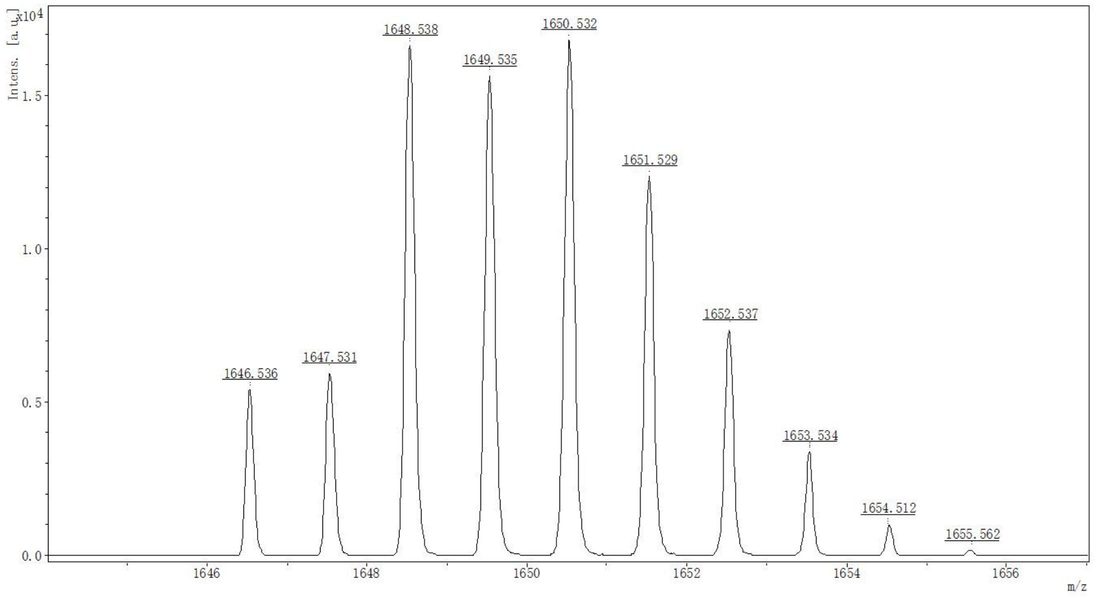

Supplementary Fig. 9 MALDI-TOF mass spectra of C11-BO-SBr.

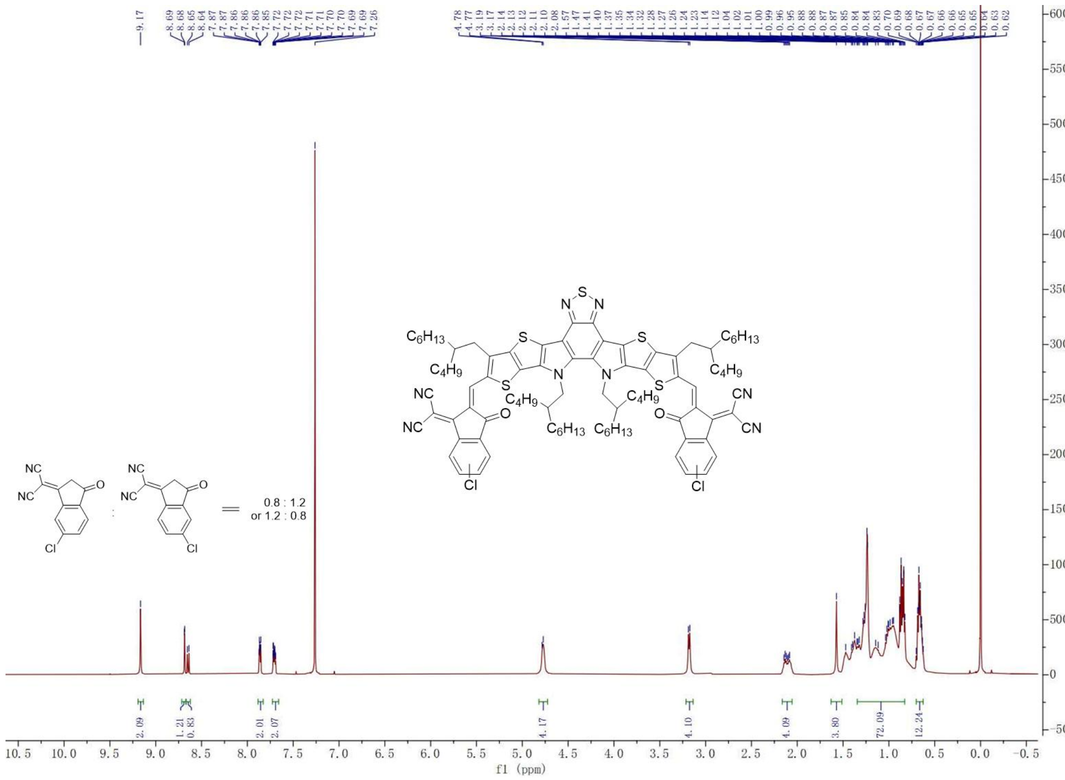

Supplementary Fig. 10 ^{1}H NMR spectrum of 2BO-SCl in $CDCl_3$.

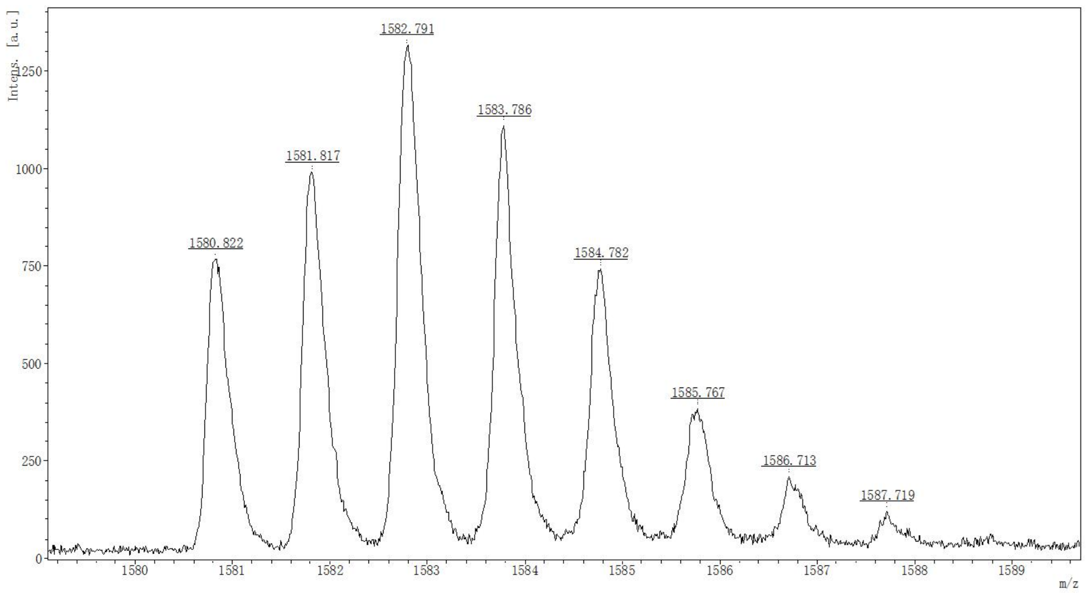

Supplementary Fig. 11 MALDI-TOF mass spectra of 2BO-SCl.

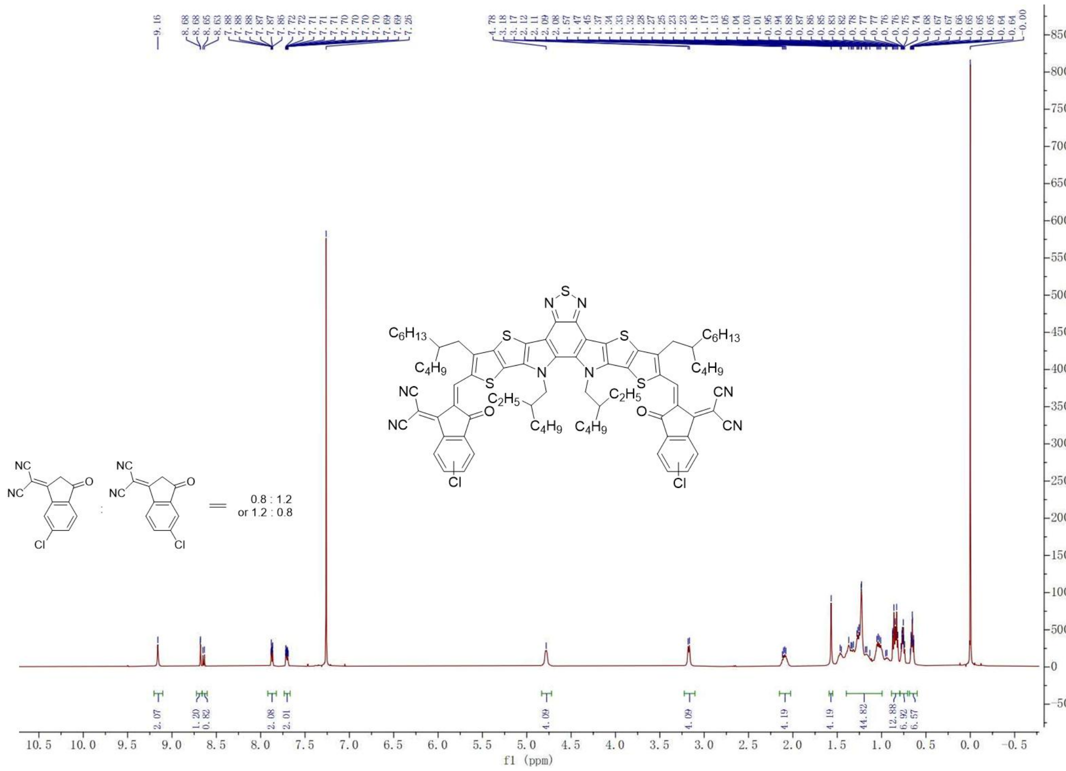

Supplementary Fig. 12 ^{1}H NMR spectrum of BO-EH-SCl in $CDCl_3$.

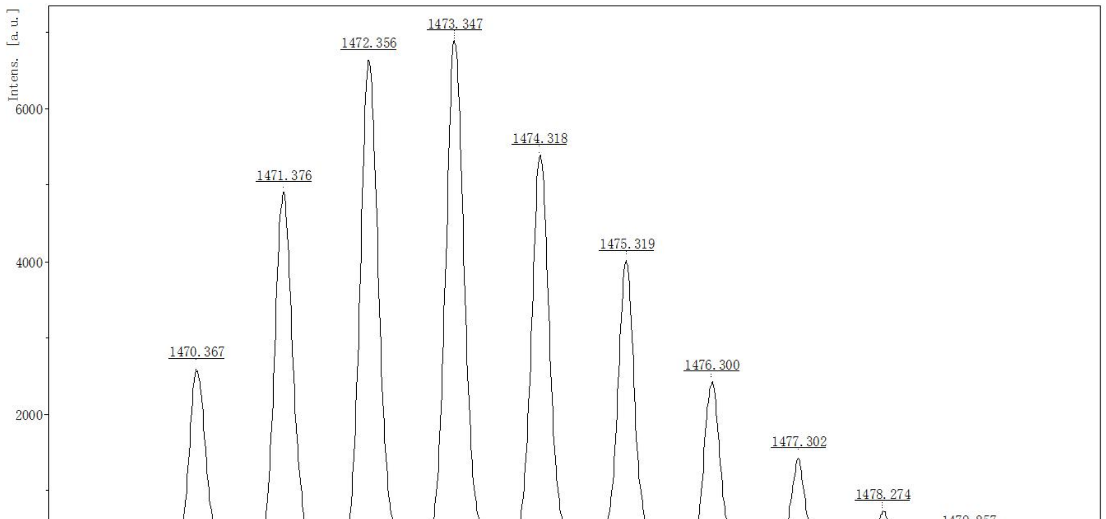

Supplementary Fig. 13 MALDI-TOF mass spectra of BO-EH-SCl.

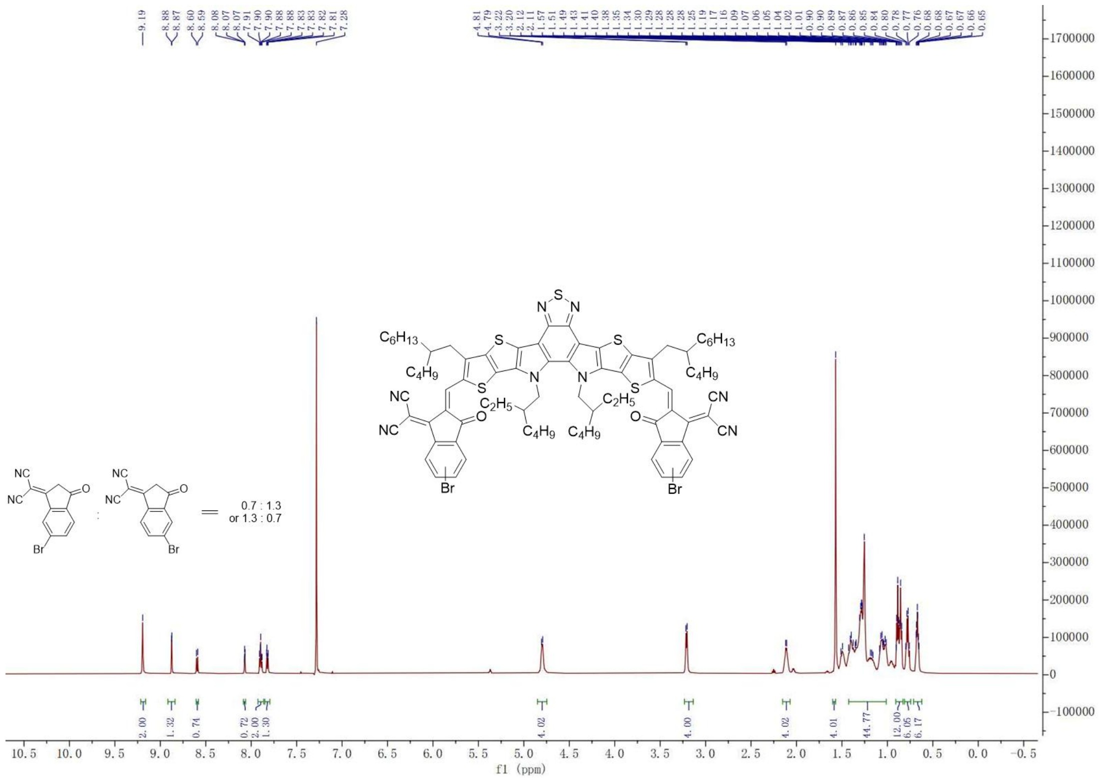

Supplementary Fig. 14 ^{1}H NMR spectrum of BO-EH-SBr in $CDCl_3$.

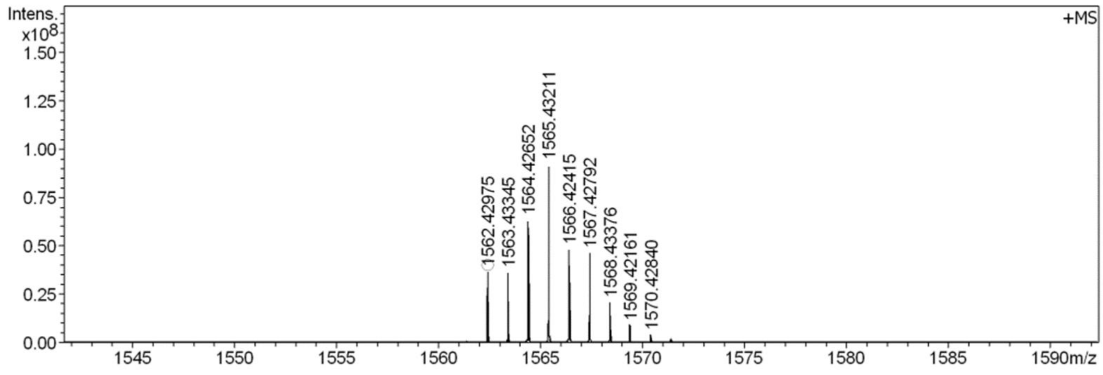

Supplementary Fig. 15 MALDI-TOF mass spectra of BO-EH-SBr.

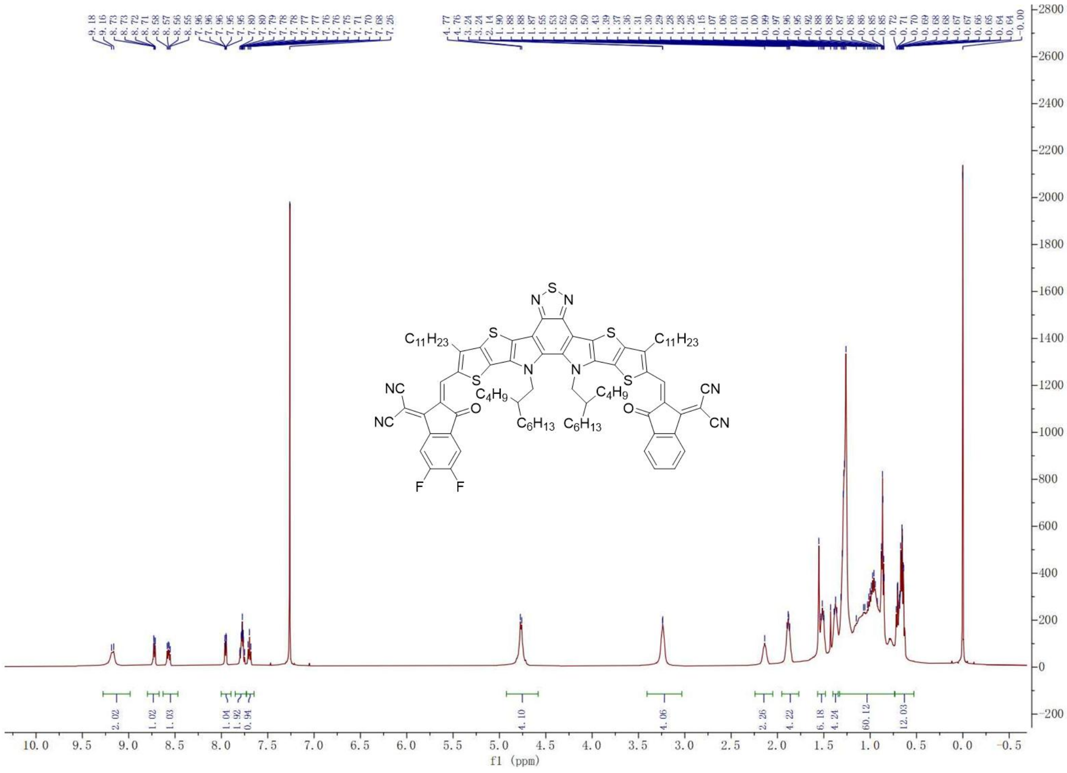

Supplementary Fig. 16 ^{1}H NMR spectrum of C11-BO-AF in $CDCl_3$.

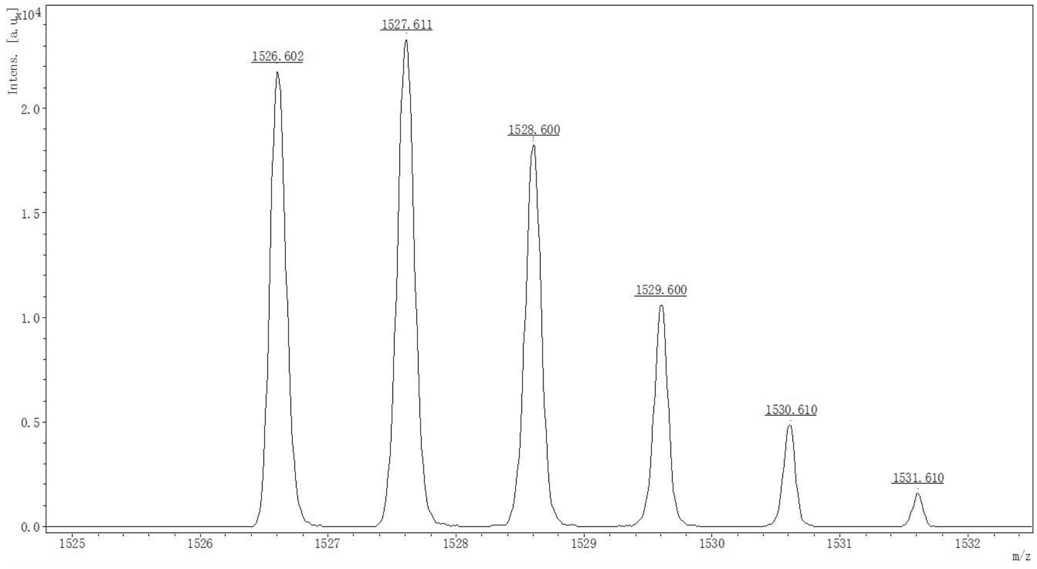

Supplementary Fig. 17 MALDI-TOF mass spectra of C11-BO-AF.

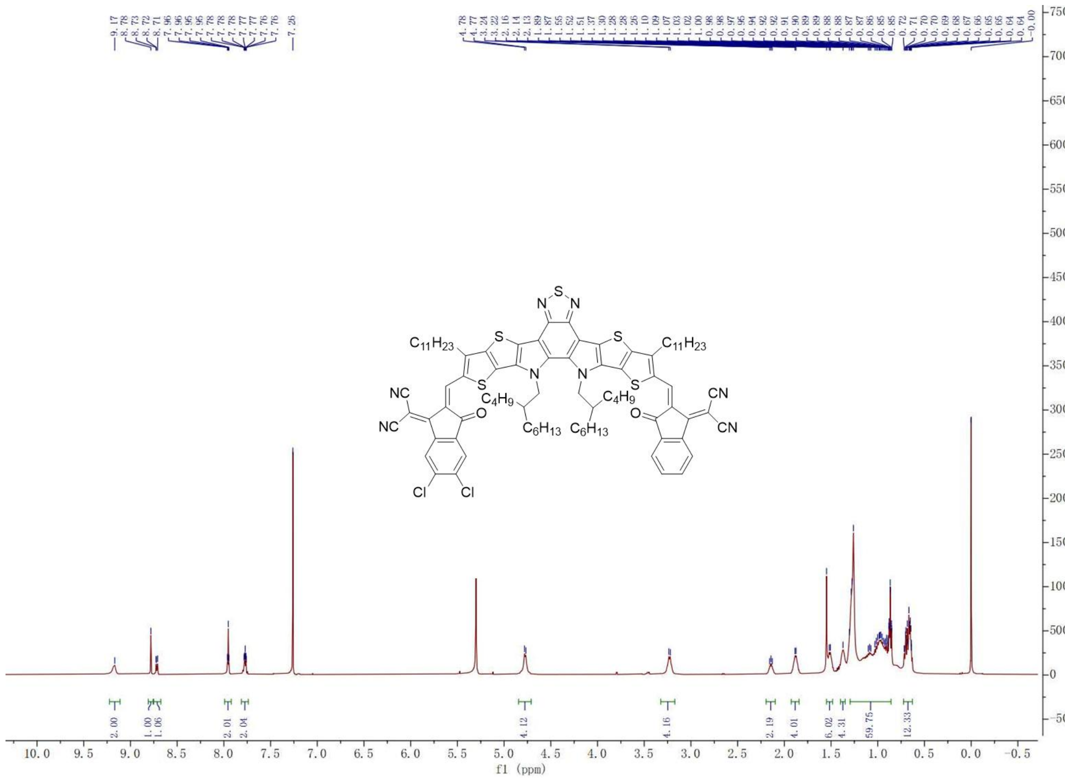

Supplementary Fig. 18 ^{1}H NMR spectrum of C11-BO-ACl in $CDCl_3$.

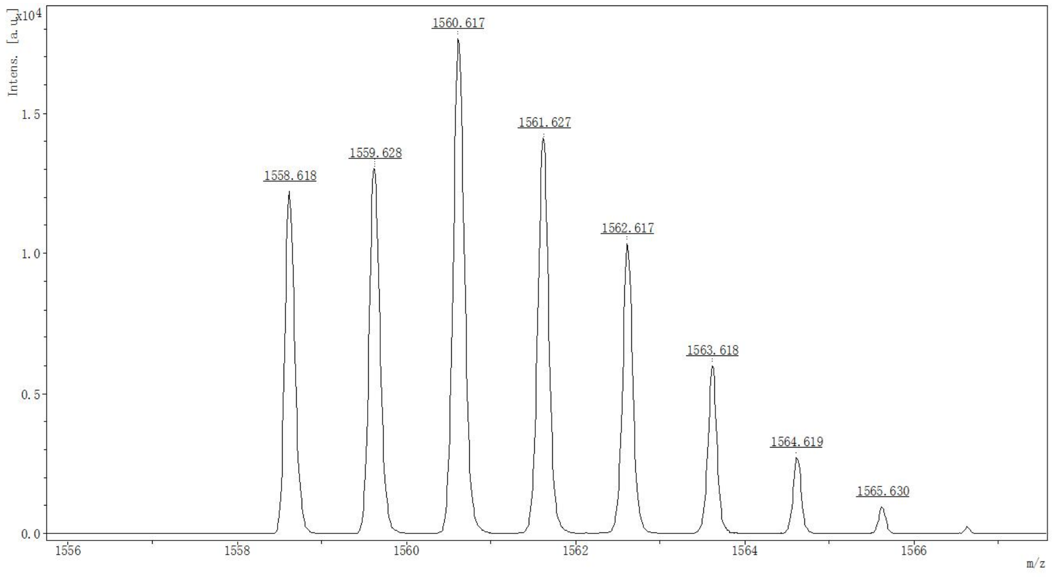

Supplementary Fig. 19 MALDI-TOF mass spectra of C11-BO-ACl.

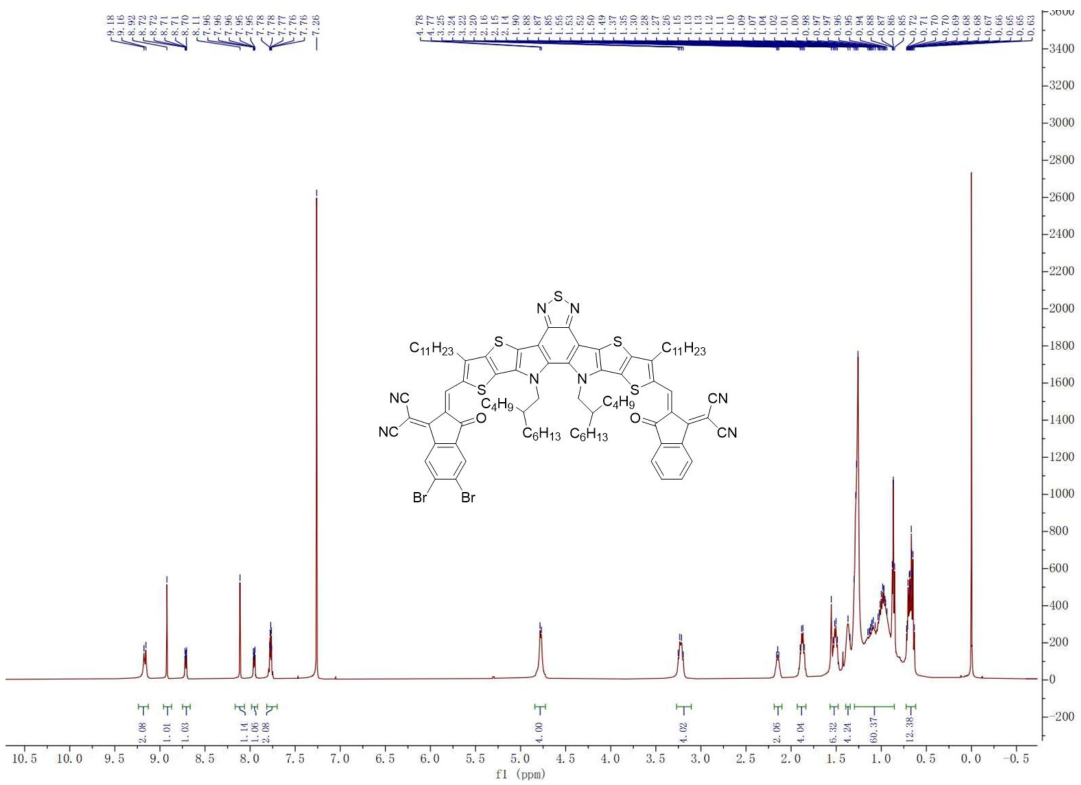

Supplementary Fig. 20 ^{1}H NMR spectrum of C11-BO-ABr in $CDCl_3$.

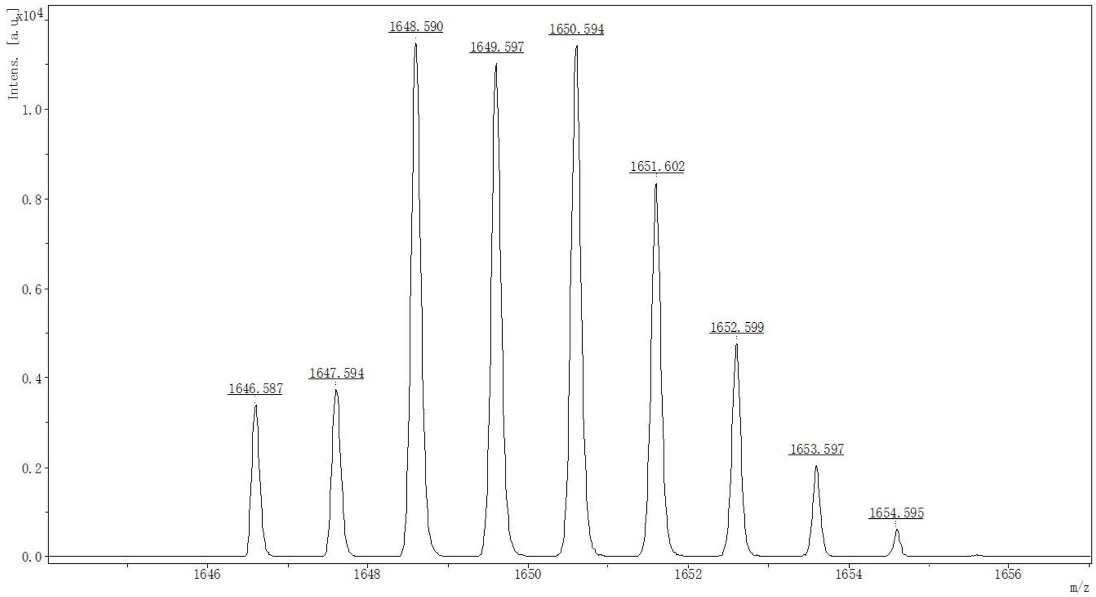

Supplementary Fig. 21 MALDI-TOF mass spectra of C11-BO-ABr.

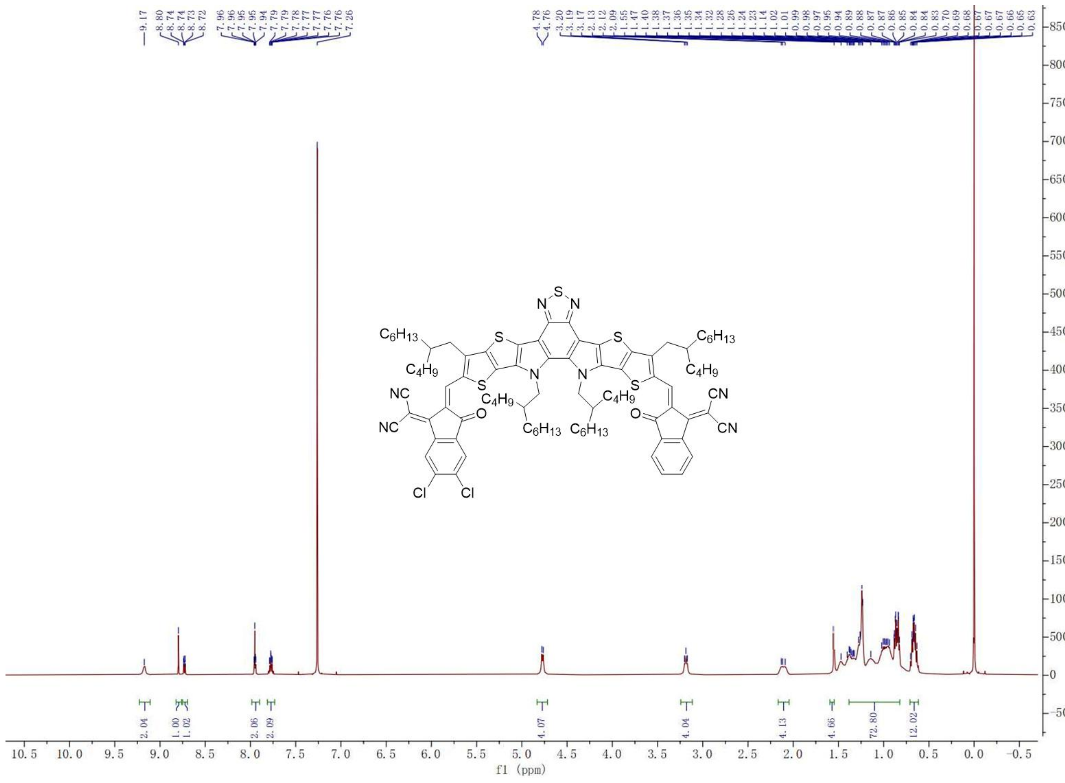

Supplementary Fig. 22 ^{1}H NMR spectrum of 2BO-ACl in $CDCl_3$.

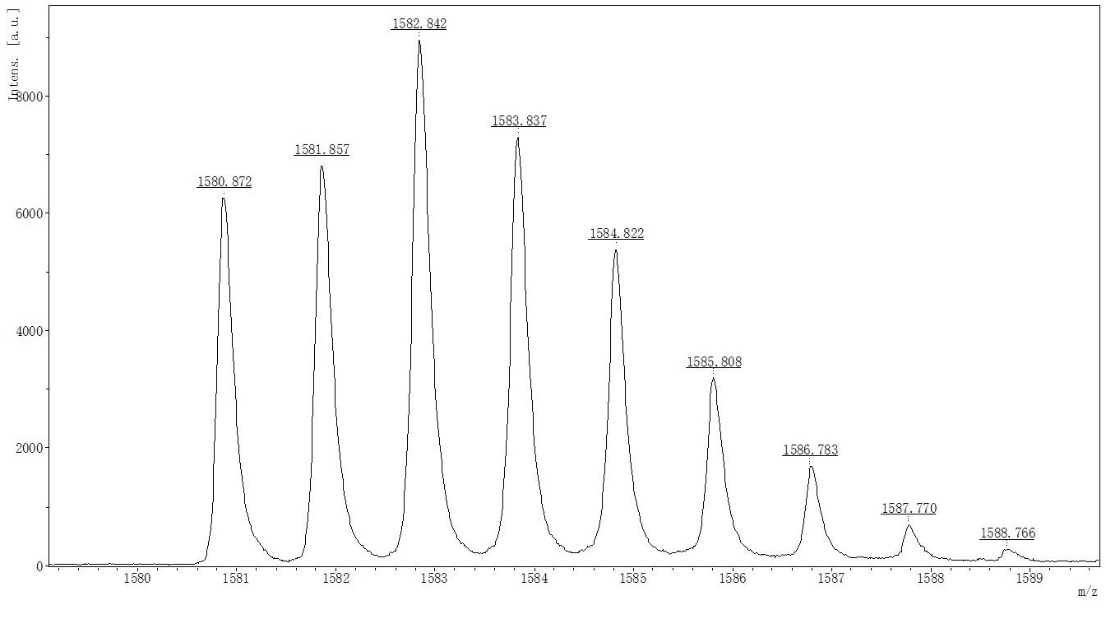

Supplementary Fig. 23 MALDI-TOF mass spectra of 2BO-ACl.

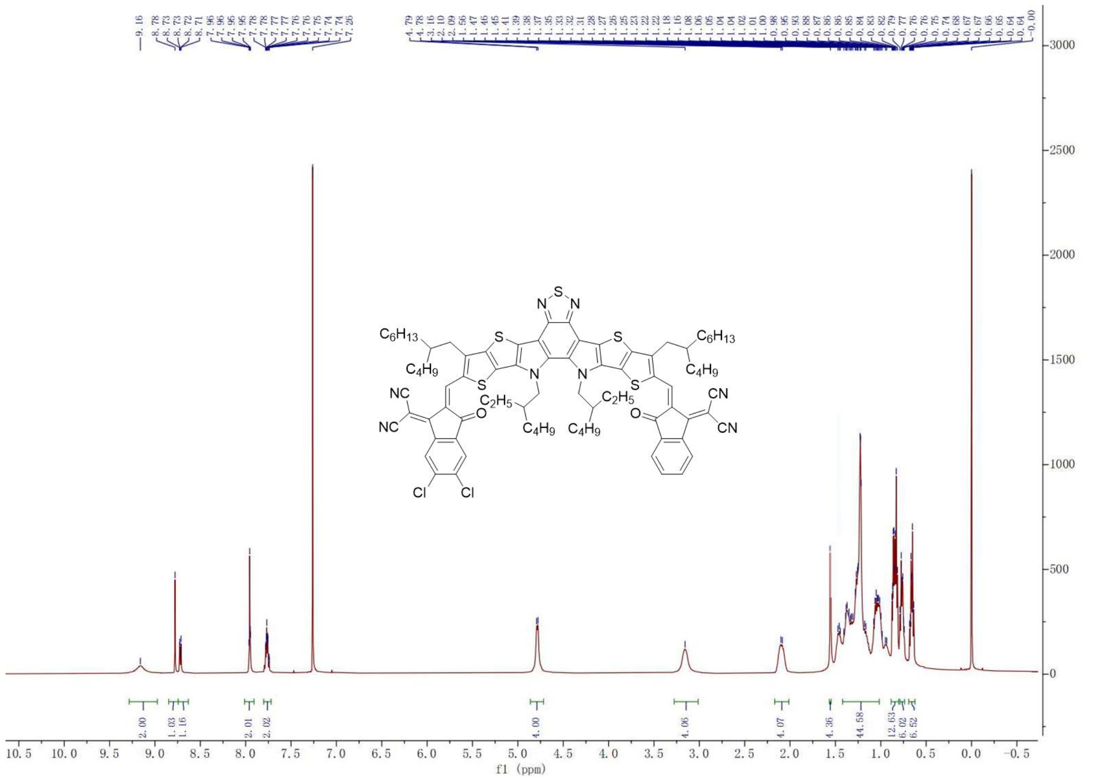

Supplementary Fig. 24 ^{1}H NMR spectrum of BO-EH-ACl in $CDCl_3$.

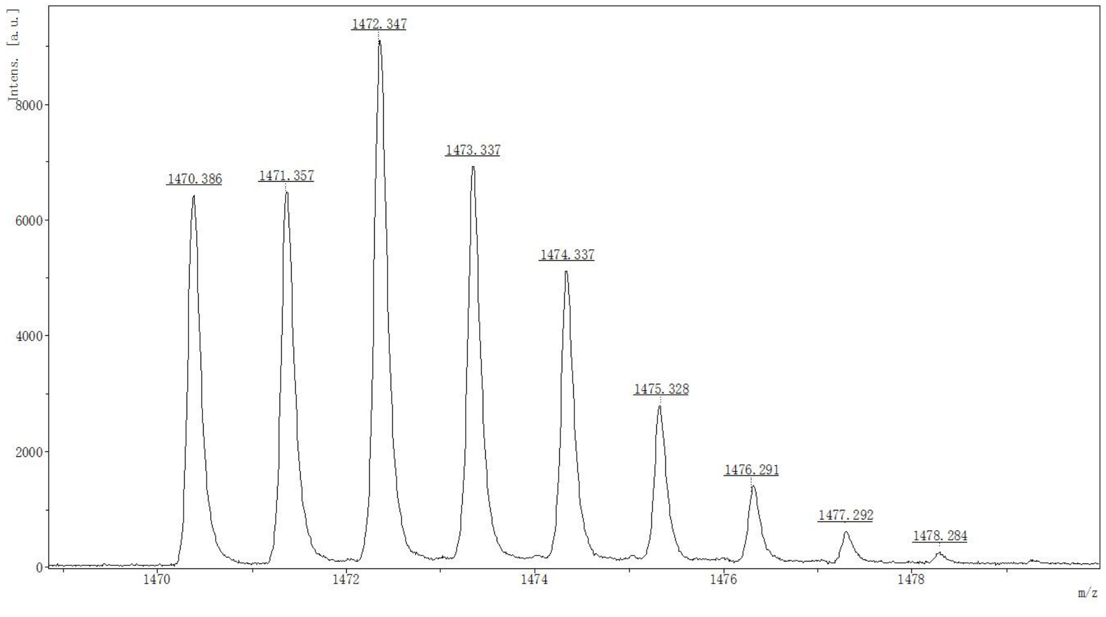

Supplementary Fig. 25 MALDI-TOF mass spectra of BO-EH-ACl.

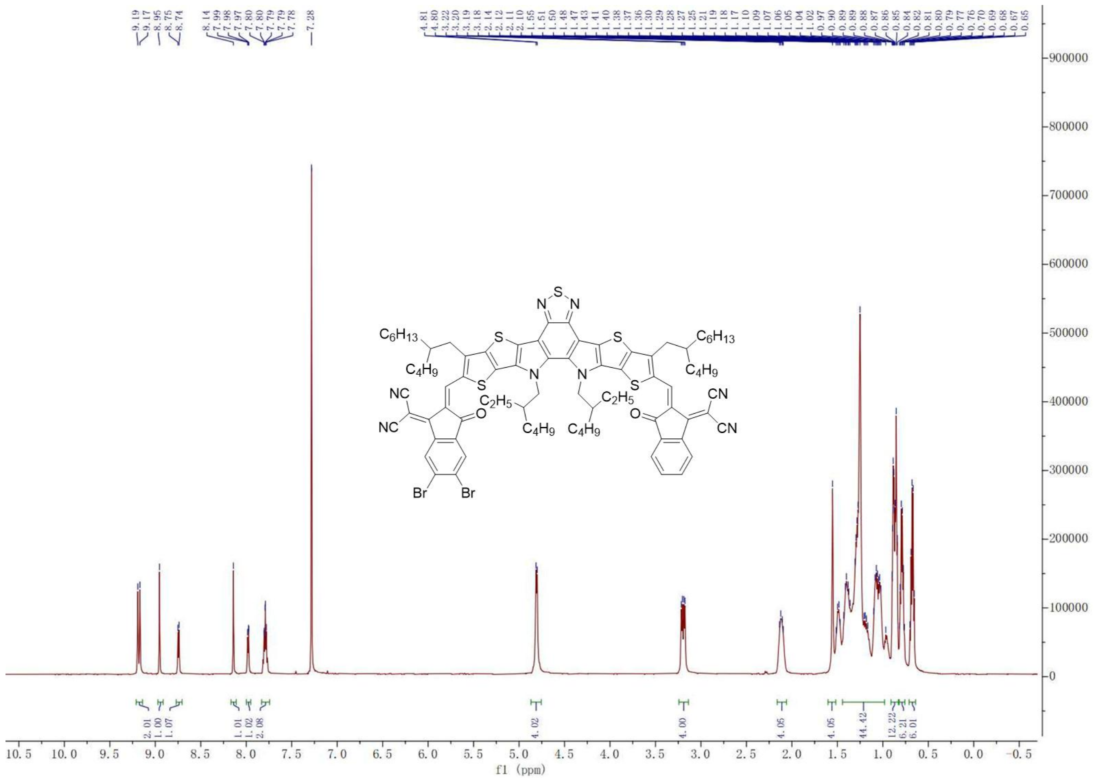

Supplementary Fig. 26 ^{1}H NMR spectrum of BO-EH-ABr in $CDCl_3$.

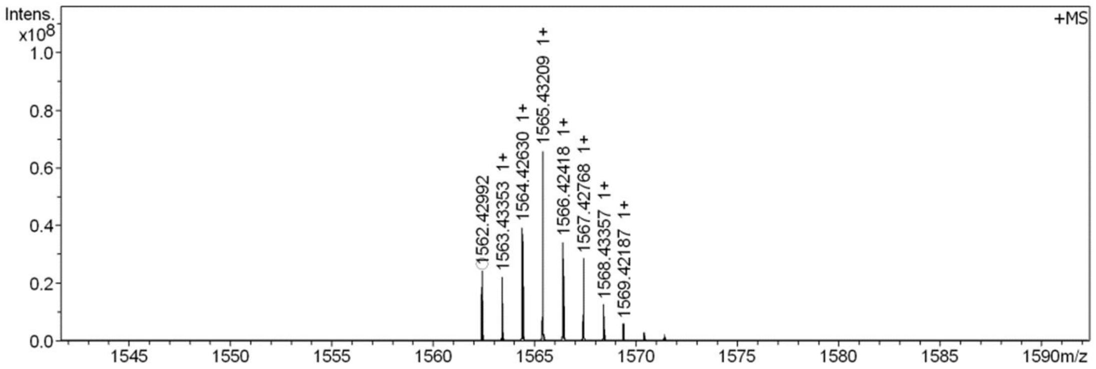

Supplementary Fig. 27 MALDI-TOF mass spectra of BO-EH-ABr.

3. Supplementary Absorption and Energy Level Measurements

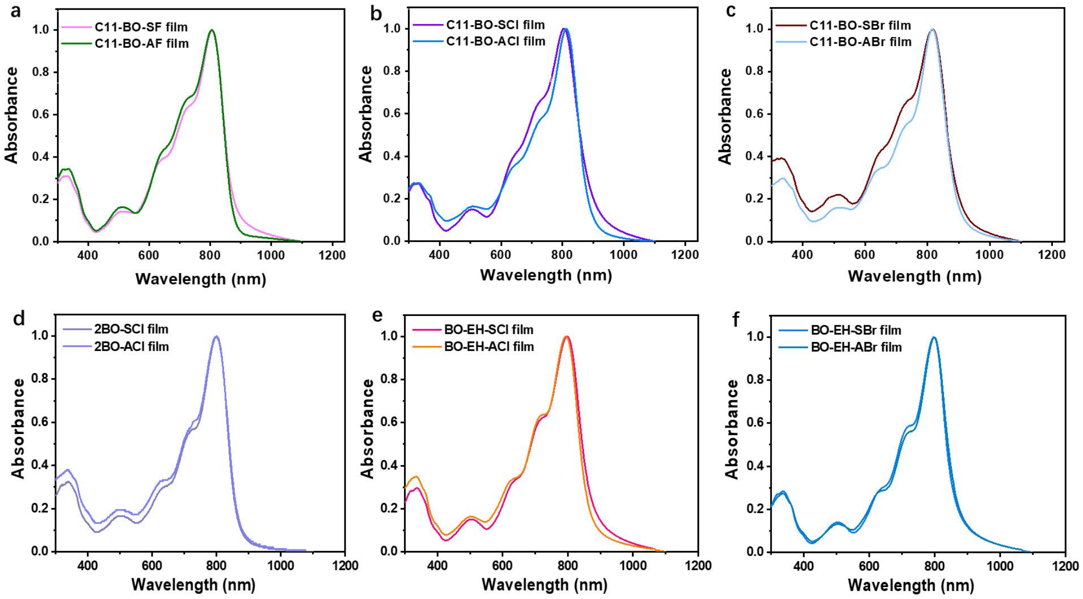

Supplementary Fig. 28 UV-vis curves of **a** C11-BO-SF and C11-BO-AF films, **b** C11-BO-SCl and C11-BO-ACl films, **c** C11-BO-SBr and C11-BO-ABr films, **d** 2BO-SCl and 2BO-ACl films, **e** BO-EH-SCl and BO-EH-ACl films and **f** BO-EH-SBr and BO-EH-ABr films.

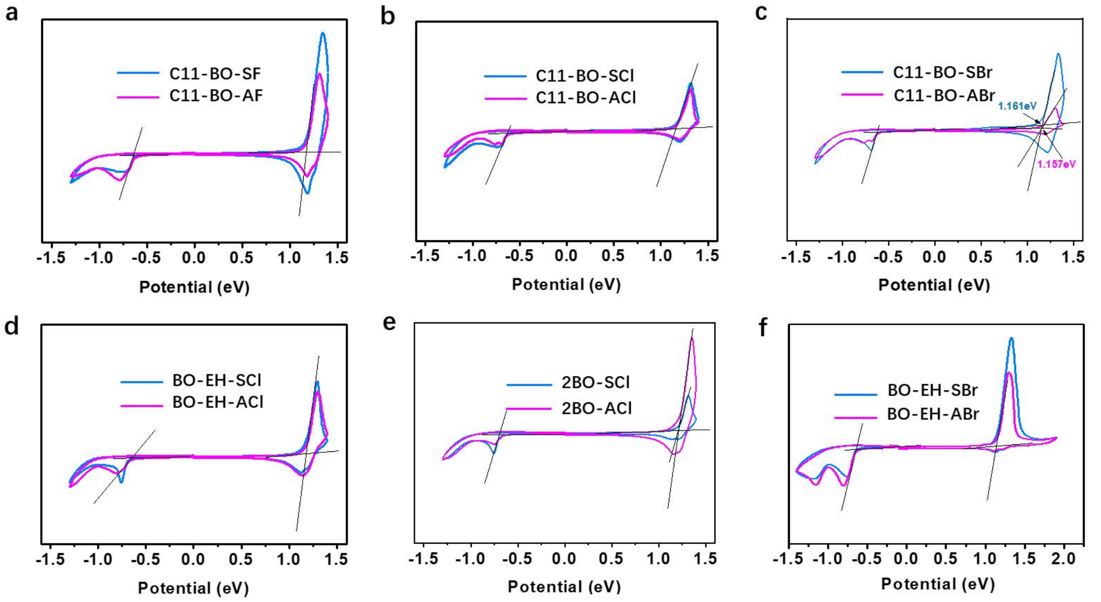

Supplementary Fig. 29 CV curves of **a** C11-BO-SF and C11-BO-AF films, **b** C11-BO-SCl and C11-BO-ACl films, **c** C11-BO-SBr and C11-BO-ABr films, **d** BO-EH-SCl and BO-EH-ACl films, **e** 2BO-SCl and 2BO-ACl films and **f**

BO-EH-SBr and BO-EH-ABr films.

4. Supplementary Morphology Characterization

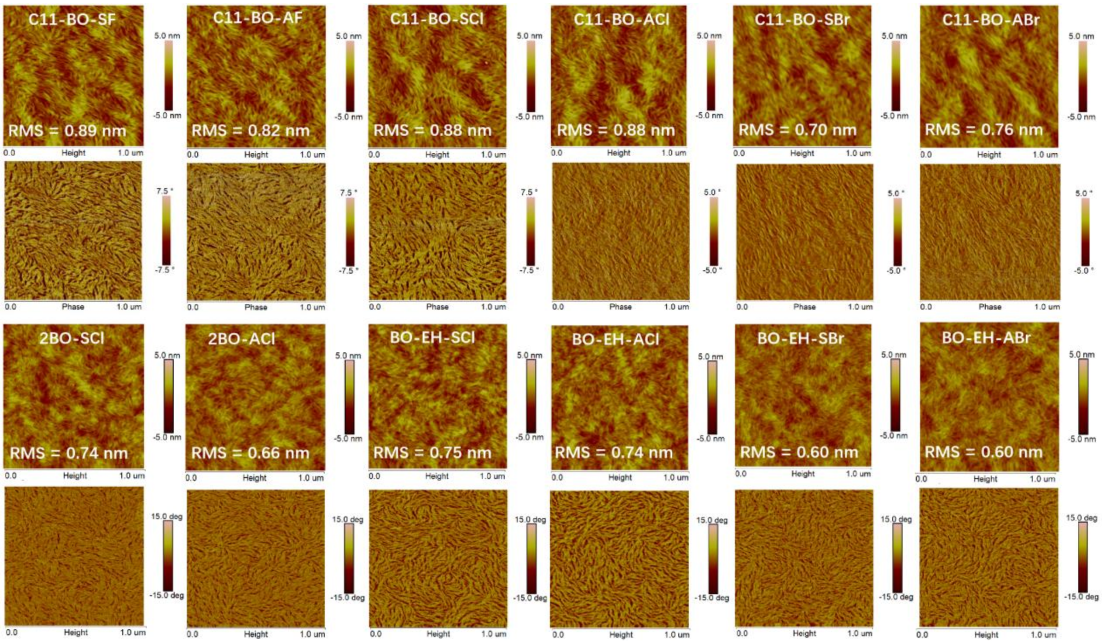

Supplementary Fig. 30 AFM height and phase images of blend films with different acceptors.

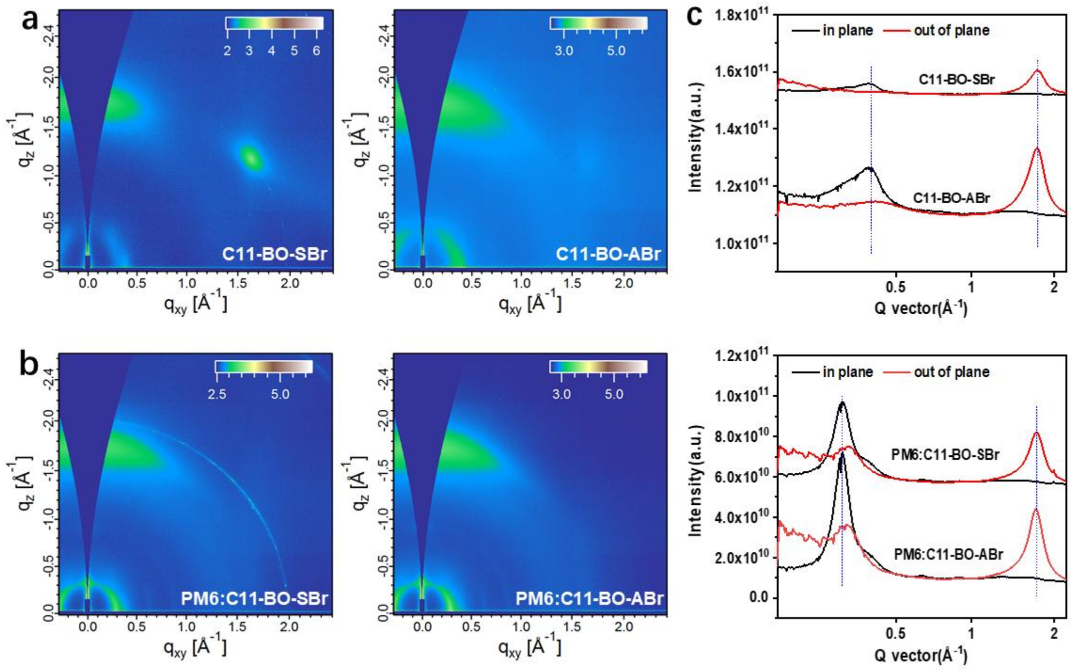

Supplementary Fig. 31 2D GIWAXS images of **a** pristine acceptor films (including BO-EH-SCl and BO-EH-ACl) and **b** blend films (including PM6:BO-EH-SCl and PM6:BO-EH-ACl). **c** GIWAXS intensity profiles of the corresponding films along the in-plane and out-of-plane directions.

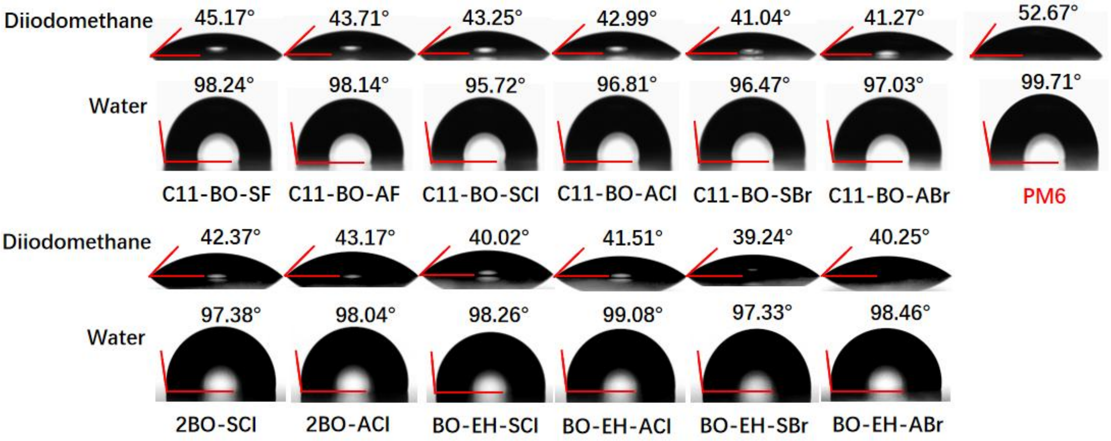

Supplementary Fig. 32 Contact angle images of PM6 and acceptors in thin films with water and diiodomethane droplet on top.

5. Supplementary Energy Loss Calculation

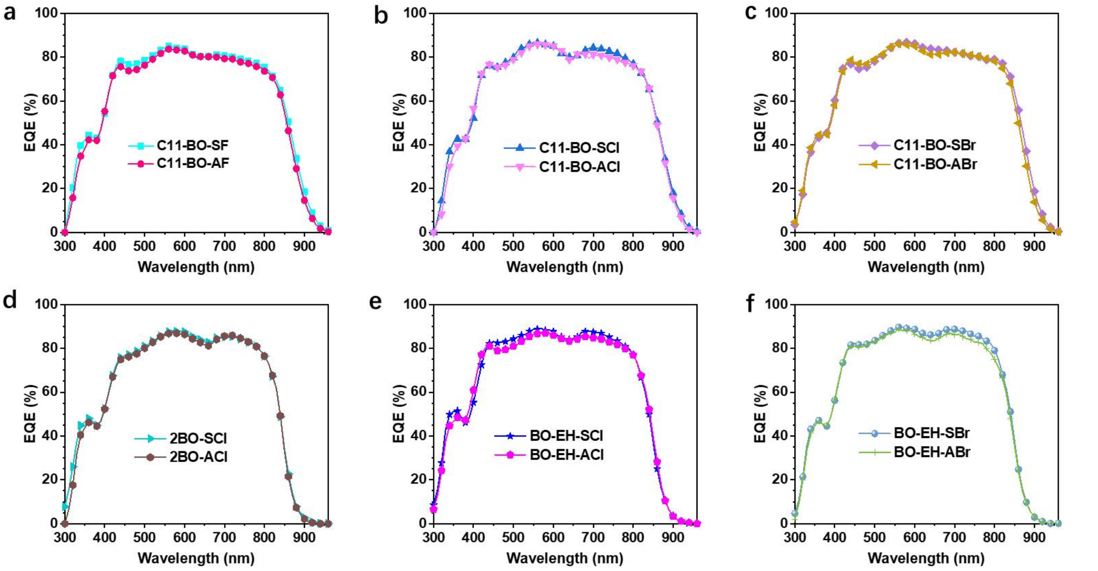

Supplementary Fig. 33 EQE curves of **a** C11-BO-SF and C11-BO-AF based films, **b** C11-BO-SCl and C11-BO-ACl based films, **c** C11-BO-SBr and C11-BO-ABr based films, **d** 2BO-SCl and 2BO-ACl based films, **e** BO-EH-SCl and BO-EH-ACl based films and **f** BO-EH-SBr and BO-EH-ABr based films.

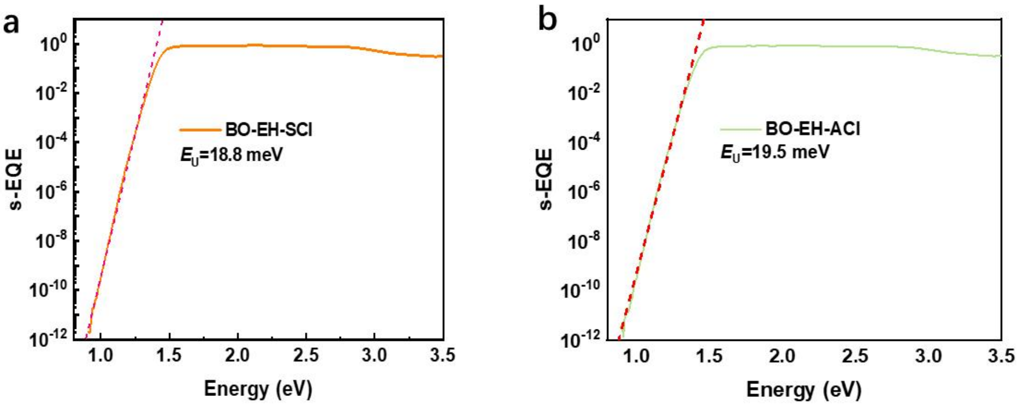

Supplementary Fig. 34 Urbach Energy (E_U) calculation based on sEQE curves of **a** BO-EH-SCl and **b** BO-EH-ACl based films.

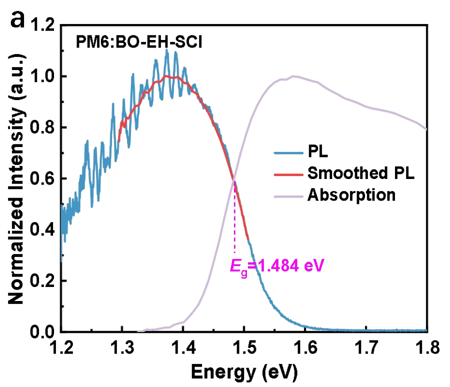

Supplementary Fig. 35 E_g determination method based on absorption curves and PL curves of **a** PM6:BO-EH-SCl and **b** PM6:BO-EH-ACl based blend films.

6. Supplementary Charge Transfer, Transport and Recombination

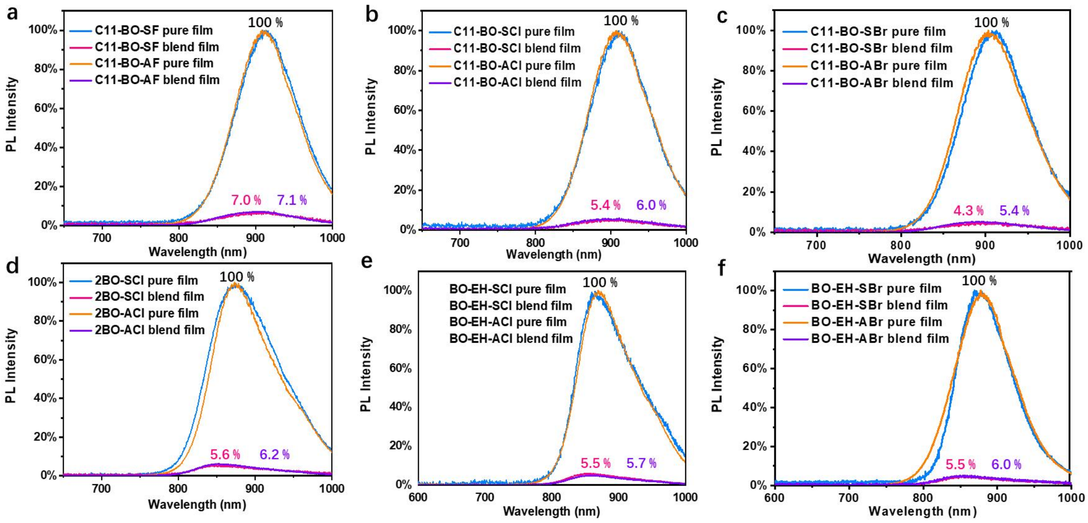

Supplementary Fig. 36 PL spectra of **a** C11-BO-SF and C11-BO-AF based pure and blend films, **b** C11-BO-SCl and C11-BO-ACl based pure and blend films, **c** C11-BO-SBr and C11-BO-ABr based pure and blend films, **d** 2BO-SCl and 2BO-ACl based pure and blend films, **e** BO-EH-SCl and BO-EH-ACl based pure and blend films and **f** BO-EH-SBr and BO-EH-ABr based pure and blend films.

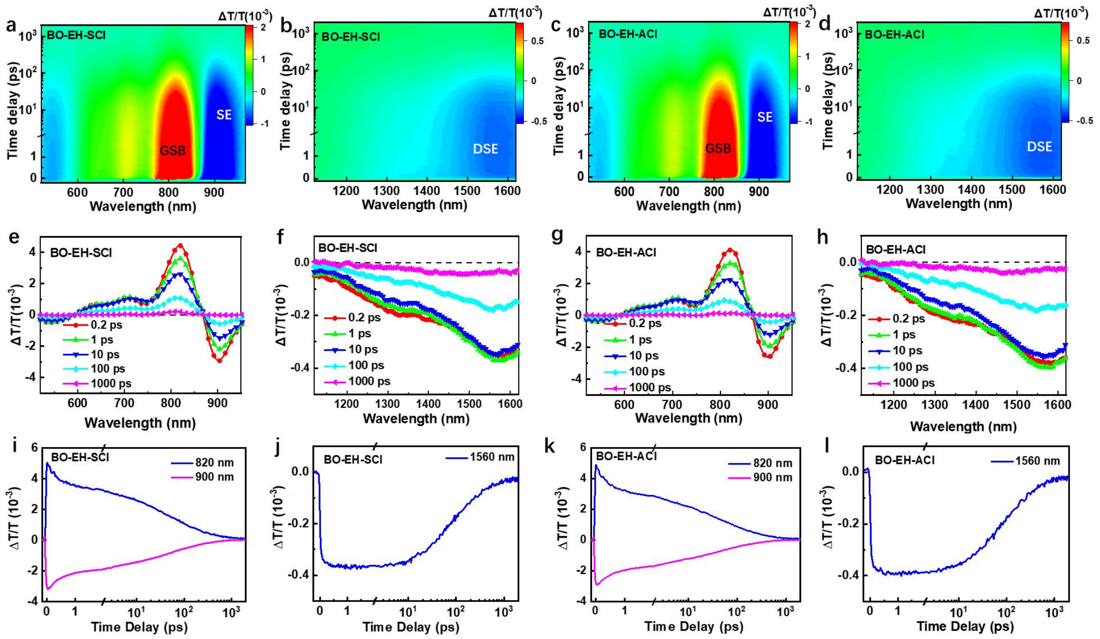

Supplementary Fig. 37 a–d 2D TAS spectra. **e–h** Color plots of the TAS spectra. **i–l** TA traces of the pure film for BO-EH-SCl and BO-EH-ACl.

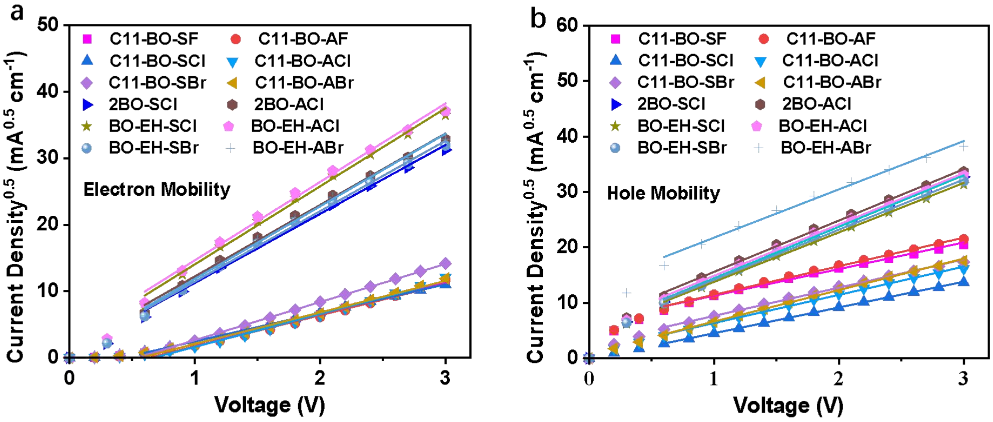

Supplementary Fig. 38 a Electron and **b** hole mobility of the optimal devices.

7. Supplementary MD Simulation

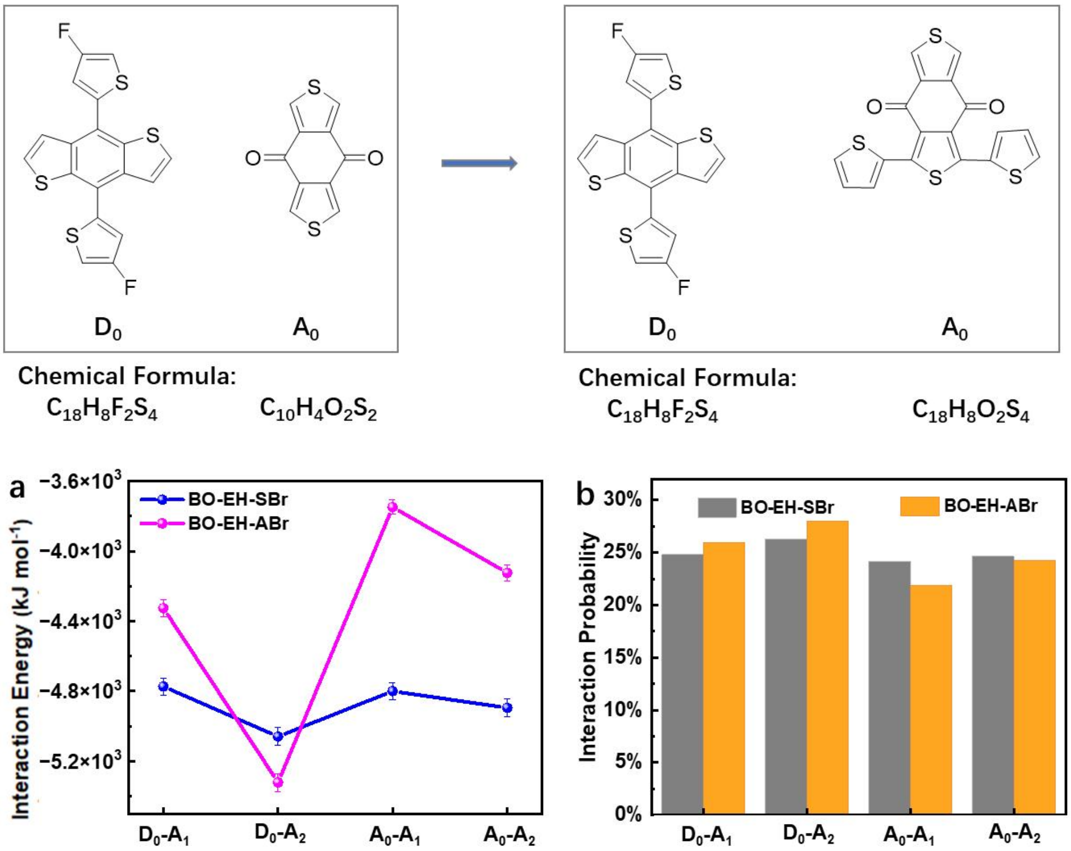

Supplementary Fig. 39 a Interaction energy, and **b** contact probability after adjusting the two fragments based on the same atom numbers.

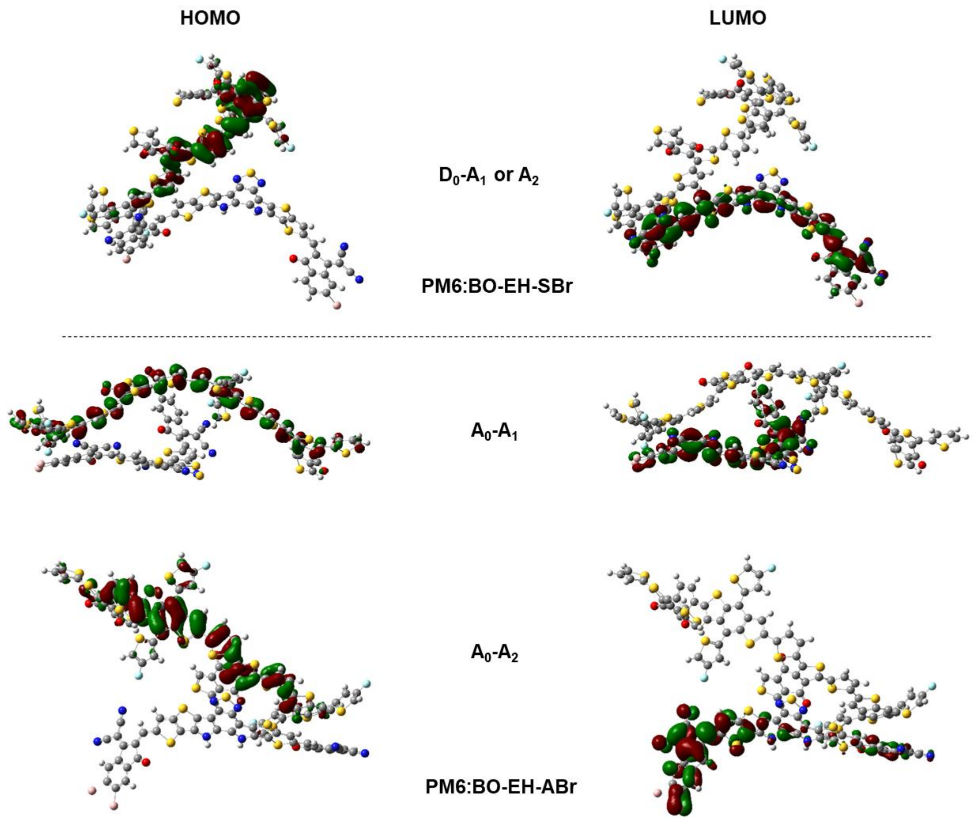

Supplementary Fig. 40 HOMO and LUMO of three typical complexes (D_0-A_1 or A_2 for PM6:BO-EH-SBr, A_0-A_1 and A_0-A_2 for PM6:BO-EH-ABr).

8. Supplementary Device Fabrication and Characterization

(1) Space Charge Limited Current (SCLC) Measurement

Hole-only devices were fabricated in a structure of ITO/PEDOT:PSS/active layer/MoO_3/Ag structure. Electron-only devices were fabricated in ITO/ZnO/active layer/PDINN/Ag structure. The device characteristics were extracted by modeling the dark current under forwarding bias using the SCLC expression described by the Mott-Gurney law:

$$J = \frac{9}{8}\varepsilon_r\varepsilon_0\mu\frac{V^2}{L^3}$$

Here, $\varepsilon_r \approx 3$ is the average dielectric constant of the blend film, ε_0 is the permittivity of the free space, μ is the carrier mobility, L is the thickness of the film and V is the applied voltage.

(2) Photoluminescence (PL) Measurement

PL spectra were detected using a home-setup microfluorescence system. The excitation light (405 nm) was generated by a semiconductor pulse diode laser centered at 405 nm (PiL1-040-40). PL spectra were measured using a spectrograph (Princeton Instruments) with a liquid-N_2-cooled CCD. Samples were kept in an N_2 filled cell at room temperature for all optical measurements.

(3) Electroluminescence External Quantum Efficiency (EQE_{EL})

A digital source meter (Keithley 2400) and a picoammeter (Keithley 6482) are used for the EQE_{EL} measurements. The former is applied to inject electric current into the solar cells to emit the photons, which will be collected using a Si diode and form electric current that can be measured by the latter.

(4) Electroluminescence (EL) Measurement

A source meter (Keithley 2400) is employed to create the injected electric current leading to the luminescence of the solar cells. After going through an optical fiber (BFL200LS02, Thorlab), the emitted light emerged from the solar cells is collected by a fluorescence spectrometer (KYMERA-3281-B2, Andor Technology) including two sets of diffraction gratings for the wavelength range of 600~1100 nm and 900~1400 nm, and is measured by a Si EMCCD camera (DU970PBVF, Andor Technology) and an InGaAs camera (DU491A-1.7, Andor Technology), respectively.

The EL spectra are corrected for the optical losses in the fibers, the spectrometer and the cameras, using a calibrated halogen lamp (HL-3P-CAL, Ocean Optics Germany GmbH).

(5) Atomic Force Microscope (AFM) Measurement

AFM images are obtained on a VeecoMultiMode atomic force microscopy in the tapping mode.

(6) Transient Absorption Spectroscopy (TAS) Measurement

The TAS measurements were performed through a Yb: KGW laser (Pharos, Light Conversion). The wavelength of fundamental output was at ~1030 nm. We used a home-built noncollinear optical parametric amplifier to generate the pump beam. The pump beam was filtered by a bandpass filter (FB800-40, Thorlabs) to selectively excite the acceptors. The pump density was kept below 2 $\mu J/cm^2$. The probe beam

was supercontinuum generated by focusing a small fraction of the fundamental 1030 beam to a 5 mm sapphire plate for visible detection or a 6 mm Yttrium Aluminum Garnet (YAG) plate for infrared detection. A short pass filter (10SWF-1000-B, Newport) or a long pass filter (FELH1100, Thorlabs) was employed in the supercontinuum to exclude the fundamental beam for visible detection (550-960 nm) or infrared detection (1100-1600 nm), respectively. The probe beam was split into two beams (probe and reference) for double-line detection. The probe and reference beams were then routed to a double-line Si camera (S14417, Hamamatsu) for visible detection or a double line InGaAs camera (G11608, Hamamatsu) for infrared detection, which is mounted on a monochromator (Acton 2358, Princeton Instrument). Pulse-to-pulse spectral analysis was conducted at the rate of 50 kHz for visible detection and 16 kHz for infrared detection enabled by homebuilt field-programmable gate array (FPGA) control boards. The noise level ($\Delta T/T$) was better than 10-5 (std) after averaging 25k pump-on and pump-off shots for each data point. The samples were kept in a nitrogen atmosphere during the measurement to prevent photon induced degradation.

(7) Grazing Incidence Wide-Angle X-ray Scattering (GIWAXS) Characterization

GIWAXS measurements were performed at beamline 7.3.3 at the Advanced Light Source. Samples were prepared on Si substrates using identical blend solutions as those used in devices. The 10 keV X-ray beam was incident at a grazing angle of 0.12°, selected to maximize the scattering intensity from the samples. The scattered X-rays were detected using a Dectris Pilatus 2M photon counting detector.

9. Supplementary Molecular Dynamics Simulations Method

The geometry optimizations calculations were performed at the long-range corrected ωB97XD/6-31G (d, p) level as **Supplementary Fig. 2**. The dielectric environment via the polarizable continuum model (PCM) was employed to tune the

range-separation parameters (ω)[1], and the dielectric constant ε value was to be 4.0 for all Density Functional Theory (DFT) calculations with Gaussian16[2]. The tuned ω values for SF, SCl, SBr, AF, ACl, and ABr are 0.0039, 0.0037, 0.0037, 0.0040, 0.0038 and 0.0038, respectively. The analysis of frontier orbitals is displayed in **Supplementary Fig. 2**, implying that intermolecular charge transfer (i-CT) exists obviously owing to the different distributions of HOMO and LUMO. Previous reports have demonstrated that CT state energy at the interfacial D: A pairs are critical to the open-circuit voltage (V_{oc}) and voltage losses, and the percentage of CT is higher than the localized excitation state[3, 4]. Moreover, the energy difference between S_1 and CT is already small in high-efficiency NFA OPVs.[5] Therefore, we calculate the nature of vertical excitation energy S_1 states to approximately characterize the CT energy.

10. Supplementary Table

Supplementary Table 1 Comparison of efficiency and V_{oc} for binary OPVs without special treatment between this work and references.

Active layer	V_{oc} [eV]	PCE [%]	
PM6:BO-EH-ACl	0.927	18.80	This work
PM6:AC9	0.871	18.43	6
PM6:L8-BO	0.87	18.32	7
PM6:L8-HD	0.88	17.32	7
PM6:BP4T-4F	0.839	17.1	8
SZ5:BPT-4F	0.853	16.5	9
SZ5:BPS-4F	0.822	16.1	9
PM6:BTP-S1	0.93	15.21	10
PM6:BTP-S2	0.95	16.37	10
PM6:Y11	0.833	16.54	11
PBDB-T: OY3	0.84	14.51	12

PM6:BO-4Cl	0.841	17.43	13
PM6:CH4	0.888	16.49	14
PM6:CH6	0.875	18.33	14
PBDB-T:DOC2C6-2F	0.85	13.24	15
PTQ10:Y6	0.87	16.21	16
PM6:SY1	0.871	16.83	17
PM6:SY2	0.852	16.01	17
PM6:SY3	0.858	16.23	17
PBT1-C:IDTT-C8-TIC	0.88	13.4	18
PM6:IQx-1	0.911	17.9	19
PM6:BTP-2F-ThCl	0.869	17.06	20
PM6:mBzS-4F	0.804	17.02	21

Supplementary Table 2 DFT, UV-vis and CV results of pure acceptors.

Acceptor	$LUMO^{DFT}$ [eV][a]	$HOMO^{DFT}$ [eV][a]	λ_{max} [nm][b]	λ_{onset} [nm][b]	λ_{max} [nm][c]	λ_{onset} [nm][c]	E_g^{opt} [eV][c]	$LUMO^{CV}$ [eV][d]	$HOMO^{CV}$ [eV][d]
C11-BO-SF	-3.45	-5.47	723	778	803	876	1.42	-3.86	-5.66
C11-BO-AF	-3.43	-5.47	722	778	802	875	1.42	-3.86	-5.66
C11-BO-SCl	-3.49	-5.51	730	788	812	898	1.38	-3.88	-5.66
C11-BO-ACl	-3.49	-5.52	731	788	810	896	1.38	-3.88	-5.66
C11-BO-SBr	-3.48	-5.50	731	788	816	902	1.37	-3.87	-5.63
C11-BO-ABr	-3.46	-5.49	731	788	818	906	1.37	-3.87	-5.63
2BO-SCl	-3.49	-5.53	728	786	801	869	1.43	-3.82	-5.67
2BO-ACl	-3.49	-5.51	730	786	800	869	1.43	-3.82	-5.67
BO-EH-SCl	-3.49	-5.53	729	786	798	878	1.41	-3.85	-5.72
BO-EH-ACl	-3.48	-5.51	730	786	793	879	1.41	-3.85	-5.72
BO-EH-SBr	-3.45	-5.47	723	778	803	876	1.42	-3.86	-5.66
BO-EH-ABr	-3.43	-5.47	722	778	802	875	1.42	-3.86	-5.66

[a]DFT calculations were performed at the b3lyp/6-31g(d) level. Absorption of the acceptors in [b]CH_3Cl solution and [c] thin films. [d]Energy levels were obtained from the CV curves.

Supplementary Table 3 Detailed data of morphology characterizations.

Acceptor	RMS [nm][a]	Diiodomethane [°][b]	Water [°][b]	γ [mN m^{-1}][b]	$\chi^{D\text{–}A}$ [b]
PM6	-	52.67	99.71	33.35	-
C11-BO-SF	0.89	45.17	98.24	37.85	0.14
C11-BO-AF	0.82	43.71	98.14	38.74	0.20
C11-BO-SCl	0.88	43.25	95.72	38.55	0.19
C11-BO-ACl	0.88	42.99	96.81	38.90	0.21
C11-BO-SBr	0.70	41.04	96.47	40.00	0.30
C11-BO-ABr	0.76	41.27	97.03	39.98	0.30
2BO-SCl	0.74	42.37	97.38	38.26	0.16
2BO-ACl	0.66	43.17	98.04	37.76	0.14
BO-EH-SCl	0.75	40.02	98.26	38.90	0.21
BO-EH-ACl	0.74	41.51	99.08	38.09	0.16
BO-EH-SBr	0.60	39.24	97.33	39.47	0.25
BO-EH-ABr	0.60	40.25	98.46	38.75	0.20

[a]RMS was derived from the AFM height images. [b]Data was summarized from the contact angle measurement.

Supplementary Table 4 Characterizations of charge transfer, transport and recombination.

Acceptor	$J_{sat.}$ [mA cm^{-2}][a]	$P_{diss.}$ [%][a]	$P_{coll.}$ [%][a]	$PL_{quench.}$ [%][b]	μ_h [cm^2 V^{-1} s^{-1}][c]	μ_e [cm^2 V^{-1} s^{-1}][c]
C11-BO-SF	25.67	97.9	97.6	93.0	4.75×10^{-4}	6.06×10^{-4}
C11-BO-AF	25.59	96.5	95.9	92.9	5.85×10^{-4}	7.31×10^{-4}
C11-BO-SCl	26.43	95.1	94.7	94.6	6.40×10^{-4}	6.89×10^{-3}
C11-BO-ACl	26.08	95.8	95.7	94.0	6.03×10^{-4}	1.09×10^{-3}
C11-BO-SBr	26.89	95.8	94.9	95.7	8.40×10^{-4}	1.13×10^{-3}

C11-BO-ABr	26.45	95.6	94.9	94.6	9.56×10^{-4}	0.92×10^{-3}
2BO-SCl	25.02	98.0	97.5	94.4	2.90×10^{-3}	3.58×10^{-3}
2BO-ACl	24.92	97.7	97.5	93.8	3.24×10^{-3}	4.14×10^{-3}
BO-EH-SCl	25.98	96.1	95.8	94.5	2.90×10^{-3}	5.08×10^{-3}
BO-EH-ACl	25.81	98.3	98.0	94.3	3.12×10^{-3}	5.25×10^{-3}
BO-EH-SBr	25.88	97.5	97.4	94.5	2.81×10^{-3}	4.03×10^{-3}
BO-EH-ABr	25.51	97.5	97.2	94.0	3.21×10^{-3}	4.33×10^{-3}

Detailed data were obtained from [a]J_{ph}–V_{eff} curves (the exciton dissociation probability ($P_{diss.}$ = J_{ph}/J_{sat}) and charge collection efficiency ($P_{coll.}$ = $J_{sc}/J_{sat.}$) of the devices were characterized under maximal power output and short circuit conditions), [b]PL spectra and [c]SCLC method.

Supplementary Table 5 Average ESP of each atom of five fragments calculated on B3LYP/6-31G(d, p) level.

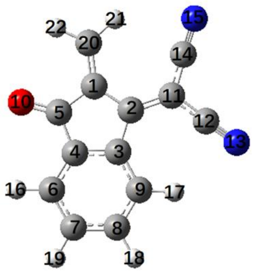

IC (or A_2)

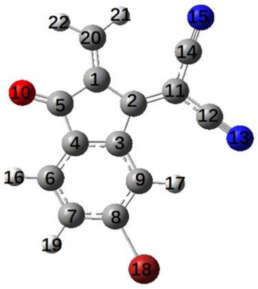

IC-Br (or A_2 = A_2)

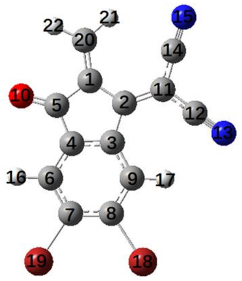

IC-2Br (or A_1)

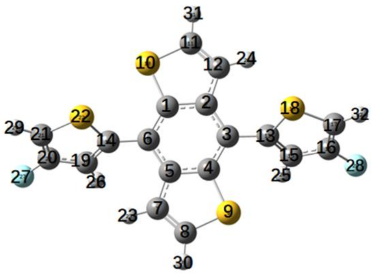

D_0 (PM6)

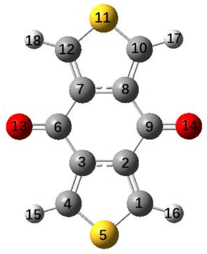

A_0 (PM6)

Atom Number	ESP(IC) [kJ mol-1]	ESP(IC-Br) [kJ mol-1]	ESP(IC-2Br) [kJ mol-1]	ESP(A_0) [kJ mol-1]	ESP(D_0) [kJ mol-1]	Atom Number	ESP(D_0) [kJ mol-1]
1	11.47	13.94	15.53	0.51	-5.11	23	6.51
2	15.26	18.07	19.60	2.02	-4.66	24	4.63
3	11.93	15.36	17.54	2.00	-2.78	25	5.63
4	10.33	13.71	15.99	0.56	-5.15	26	8.47
5	13.17	15.71	17.70	6.14	-4.71	27	-8.00
6	6.72	10.19	12.33	4.63	-2.64	28	-8.59
7	7.04	10.20	11.50	2.04	-5.90	29	10.68
8	6.67	9.05	11.14	2.00	-5.94	30	11.74
9	7.20	10.46	12.83	4.56	-4.80	31	11.50
10	-15.48	-13.35	-11.87	0.52	-5.75	32	11.03
11	12.78	15.35	16.67	6.14	-6.48		
12	6.65	8.52	9.64	0.58	-6.48		
13	-17.32	-16.07	-15.10	-21.45	-4.49		
14	6.27	8.78	10.05	-21.52	-4.54		
15	-17.90	-16.38	-15.28	10.29	-4.37		
16	12.98	16.18	14.04	10.26	-3.07		
17	10.61	8.48	10.12	10.30	-4.83		
18	19.11	2.84	3.35	10.32	-0.77		
19	20.19	18.53	4.83		-4.54		
20	7.49	9.75	11.22		-2.21		
21	10.12	12.20	13.13		-5.27		
22	9.66	11.67	13.54		-2.35		

11. Supplementary References

1. Korzdorfer, T. *et al.* Organic electronic materials: recent advances in the DFT description of the ground and excited states using tuned range-separated hybrid functionals. *Acc. Chem. Res.* **47**, 3284 (2014).

2. Frisch, M. J. *et al.* D. J. *Gaussian 16 Rev. C.01*, Wallingford, CT (2016).

3. Vandewal, K. *et al.* On the origin of the open-circuit voltage of polymer–fullerene solar cells. *Nat. mater.* **8**, 904–909(2009).

4. Yang, J. *et al.* Theoretical study of non-fullerene acceptors using end-capped groups with different electron-withdrawing abilities toward efficient organic solar cells. *J. Phys. Chem. Lett.* **13**, 916–922 (2022).

5. Chen, Z. *et al.* Triplet exciton formation for non-radiative voltage loss in high-efficiency nonfullerene organic solar cells. *Joule* **5**, 1832–1844 (2021).

6. He, C. *et al.* Compromising Charge Generation and Recombination with

Asymmetric Molecule for High-Performance Binary Organic Photovoltaics with Over 18% Certified Efficiency. *Adv. Funct. Mater.* **32**, 2112511 (2022).

7. Li, C. *et al.* Non-fullerene acceptors with branched side chains and improved molecular packing to exceed 18% efficiency in organic solar cells. *Nat. Energy* 6, 605–613 (2021).

8. Gao, W. *et al.* Asymmetric Acceptors Enabling Organic Solar Cells to Achieve an over 17% Efficiency: Conformation Effects on Regulating Molecular Properties and Suppressing Nonradiative Energy Loss. *Adv. Energy Mater.* **11**, 2003177 (2020).

9. Chai, G. *et al.* Deciphering the Role of Chalcogen-Containing Heterocycles in Nonfullerene Acceptors for Organic Solar Cells. *ACS Energy Lett.* **5**, 3415–3425 (2020).

10. Li, S. *et al.* Asymmetric Electron Acceptors for High-Efficiency and Low-Energy-Loss Organic Photovoltaics. *Adv. Mater.* **32**, 2001160 (2020).

11. Liu, S. *et al.* High-efficiency organic solar cells with low non-radiative recombination loss and low energetic disorder. *Nat. Photonics* 14, 300–305 (2020).

12. Liang, Y. *et al.* Organic solar cells using oligomer acceptors for improved stability and efficiency. *Nat. Energy* **7**, 1180–1190 (2022).

13. Chen, H. *et al.* Asymmetric electron acceptor enables highly luminescent organic solar cells with certified efficiency over 18%. *Nat. Commun.* **13**, 2598 (2020).

14. Chen, H. *et al.* Central Unit Fluorination of Non-Fullerene Acceptors Enables Highly Efficient Organic Solar Cells with Over 18 % Efficiency. *Angew. Chem. Int. Ed.* **61**, 202209580 (2022).

15. Huang, H. *et al.* Noncovalently fused-ring electron acceptors with near-infrared absorption for high-performance organic solar cells. *Nat. Commun.* **10**, 3038 (2019).

16. Yang, W. *et al.* Balancing the efficiency, stability, and cost potential for organic solar cells via a new figure of merit. *Joule* **5**, 1209–1230 (2021).

17. Liu, T. *et al.* Asymmetric Acceptors with Fluorine and Chlorine Substitution for Organic Solar Cells toward 16.83% Efficiency. *Adv. Funct. Mater.* **30**, 2000456 (2020).

18. Ye, L. *et al.* Unraveling the influence of non-fullerene acceptor molecular

packing on photovoltaic performance of organic solar cells. *Nat. Commun.* **11**, 6005 (2020).

19. Shi, Y. *et al.* Small reorganization energy acceptors enable low energy losses in non-fullerene organic solar cells. *Nat. Commun.* **13**, 3256 (2022).

20. Luo, Z. *et al.* Fine-Tuning Energy Levels via Asymmetric End Groups Enables Polymer Solar Cells with Efficiencies over 17%. *Joule* **4**, 1236–1247 (2020).

21. Qi, F. *et al.* Over 17% Efficiency Binary Organic Solar Cells with Photoresponses Reaching 1000 nm Enabled by Selenophene-Fused Nonfullerene Acceptors. *ACS Energy Lett.* **6**, 9–15 (2020).
