# Supplementary material for: On the role of asymmetric molecular geometry in high-performance organic solar cells
Source: Nat Commun. 2024 Apr 16;15:3287. doi: 10.1038/s41467-024-47707-5 (PMC11021434; doi:10.1038/s41467-024-47707-5)
Supplement: Supplementary file 3 — Reporting Summary [file 41467_2024_47707_MOESM3_ESM.pdf]

Corresponding author(s): Xian-Kai Chen, Hongzheng Chen, Lijian Zuo

# Solar Cells Reporting Summary

Nature Portfolio wishes to improve the reproducibility of the work that we publish. This form is intended for publication with all accepted papers reporting the characterization of photovoltaic devices and provides structure for consistency and transparency in reporting. Some list items might not apply to an individual manuscript, but all fields must be completed for clarity.

For further information on Nature Research policies, including our data availability policy, see Authors & Referees.

## Experimental design

**Please check the following details are reported in the manuscript, and provide a brief description or explanation where applicable.**

### 1. Dimensions

Area of the tested solar cells

- [x] Yes — A mask with area of 4.572 mm2 (certified by National Industry Metrology and Testing Center, China) was used for test.
- [ ] No — *Explain why this information is not reported/not relevant.*

Method used to determine the device area

- [x] Yes — The device was completed with an active area of 6 mm2, as defined by the overlapping area of ITO and Ag, described in the section of "Methods" in the Manuscript.
- [ ] No — *Explain why this information is not reported/not relevant.*

### 2. Current-voltage characterization

Current density-voltage (J-V) plots in both forward and backward direction

- [ ] Yes
- [x] No — Organic solar cells do not have hysteresis problems. The devices were scanned in forward direction.

Voltage scan conditions

- [ ] Yes — *Provide a description of the measurement conditions (e.g. scan direction, speed, dwell times).*
- [x] No — Due to the weak impact of voltage scan conditions on device performances, we did not provide the specific information.

Test environment

- [x] Yes — Devices were tested at room temperature (ca. 25 Celsius degree) in N2-filled glove box, described in the section of "Methods" in the Manuscript.
- [ ] No — *Explain why this information is not reported/not relevant.*

Protocol for preconditioning of the device before its characterization

- [ ] Yes — *Provide a description of the protocol.*
- [x] No — No preconditioning protocol.

Stability of the J-V characteristic

- [ ] Yes — *Provide a description of the method used. The stability of the J-V characteristic can be verified with time evolution of the maximum power point or with the photocurrent at maximum power point; see ref. 5 for details.*
- [x] No — MPP tracking is not necessary for organic solar cells.

### 3. Hysteresis or any other unusual behaviour

Description of the unusual behaviour observed during the characterization

- [ ] Yes — *Provide a description of hysteresis or any other unusual behaviour observed during the characterization.*
- [x] No — No hysteresis or other unusual behavior was observed during the characterization of the devices.

Related experimental data

- [ ] Yes — *Provide a description of the related experimental data.*
- [x] No — No hysteresis or other unusual behavior was observed during the characterization of the devices.

### 4. Efficiency

External quantum efficiency (EQE) or incident photons to current efficiency (IPCE)

- [x] Yes — The EQE data were measured by a Solar Cell Spectral Response Measurement System (RE-R, Enlitech).
- [ ] No — *Explain why this information is not reported/not relevant.*

| | | |
|---|---|---|
| A comparison between the integrated response under the standard reference spectrum and the response measure under the simulator | [x] Yes<br>[ ] No | The integrated Jcal values from EQE spectra are consistent with Jsc values from J-V measurements (Figure 2 and Table 1).<br>*Explain why this information is not reported/not relevant.* |
| For tandem solar cells, the bias illumination and bias voltage used for each subcell | [ ] Yes<br>[x] No | *Provide a description of the measurement conditions.*<br>Only single-junction solar cells. |

5. Calibration

| | | |
|---|---|---|
| Light source and reference cell or sensor used for the characterization | [x] Yes<br>[ ] No | Described in the section of "Methods" in the Manuscript.<br>*Explain why this information is not reported/not relevant.* |
| Confirmation that the reference cell was calibrated and certified | [x] Yes<br>[ ] No | Described in the section of "Methods" in the Manuscript.<br>*Explain why this information is not reported/not relevant.* |
| Calculation of spectral mismatch between the reference cell and the devices under test | [x] Yes<br>[ ] No | The light spectrum used for measurements matches well with the reference silicon cell, and we did not calculate the spectral mismatch between the reference cell and the tested devices.<br>*Explain why this information is not reported/not relevant.* |

6. Mask/aperture

| | | |
|---|---|---|
| Size of the mask/aperture used during testing | [x] Yes<br>[ ] No | A mask with area of 4.572 mm2 (certified by National Industry Metrology and Testing Center, China) was used for test.<br>*Explain why this information is not reported/not relevant.* |
| Variation of the measured short-circuit current density with the mask/aperture area | [ ] Yes<br>[x] No | *Report the difference in the short-circuit current density values measured with the mask and aperture area.*<br>All the devices were tested with a mask. |

7. Performance certification

| | | |
|---|---|---|
| Identity of the independent certification laboratory that confirmed the photovoltaic performance | [ ] Yes<br>[x] No | *Identify the independent certification laboratory.*<br>Since our work did not create the highest efficiency, we did not apply for the certification reports. |
| A copy of any certificate(s) | [ ] Yes<br>[x] No | *Certificate copies should be provided in the Supplementary information. Please state the supplementary item number.*<br>Since our work did not create the highest efficiency, we did not apply for the certification reports. |

8. Statistics

| | | |
|---|---|---|
| Number of solar cells tested | [x] Yes<br>[ ] No | Number of solar cells tested is provided in Table 1.<br>*Explain why this information is not reported/not relevant.* |
| Statistical analysis of the device performance | [x] Yes<br>[ ] No | Statistical analysis is provided in Figure 2 and Table 1.<br>*Explain why this information is not reported/not relevant.* |

9. Long-term stability analysis

| | | |
|---|---|---|
| Type of analysis, bias conditions and environmental conditions | [ ] Yes<br>[x] No | *Provide a description of the type of analysis, bias conditions and environmental conditions (e.g. illumination type, temperature, atmosphere humidity, encapsulation method, preconditioning temperature, bias) for each long-term stability analysis carried out; see ref. 7 and 8 for details.*<br>As we mainly focus on structure-properties relationship analysis, we have not done stability measurements on our devices. |
